# Stable Membrane-Association of mRNAs in Etiolated, Greening and Mature Plastids

**DOI:** 10.3390/ijms18091881

**Published:** 2017-08-31

**Authors:** Julia Legen, Christian Schmitz-Linneweber

**Affiliations:** Institut of Biology, Department of the Life Sciences, Humboldt-Universität Berlin, Philippstraße 11–13, Grüne Amöbe, 10115 Berlin, Germany; legenjul@staff.hu-berlin.de

**Keywords:** chloroplast, etioplast, membrane, organelle, ribosome, RNA processing, translation, *Zea mays*

## Abstract

Chloroplast genes are transcribed as polycistronic precursor RNAs that give rise to a multitude of processing products down to monocistronic forms. Translation of these mRNAs is realized by bacterial type 70S ribosomes. A larger fraction of these ribosomes is attached to chloroplast membranes. This study analyzed transcriptome-wide distribution of plastid mRNAs between soluble and membrane fractions of purified plastids using microarray analyses and validating RNA gel blot hybridizations. To determine the impact of light on mRNA localization, we used etioplasts, greening plastids and mature chloroplasts from *Zea mays* as a source for membrane and soluble extracts. The results show that the three plastid types display an almost identical distribution of RNAs between the two organellar fractions, which is confirmed by quantitative RNA gel blot analyses. Furthermore, they reveal that different RNAs processed from polycistronic precursors show transcript-autonomous distribution between stroma and membrane fractions. Disruption of ribosomes leads to release of mRNAs from membranes, demonstrating that attachment is likely a direct consequence of translation. We conclude that plastid mRNA distribution is a stable feature of different plastid types, setting up rapid chloroplast translation in any plastid type.

## 1. Introduction

Subcellular RNA localization is an important means of gene regulation in eukaryotic organisms [1], but also in bacteria [2]. In plants, insights into intracellular RNA localization are limited and have been mostly studied for the localization of mRNAs for endosperm storage proteins [3]. Plant cells do not only contain RNAs in the nucleo-cytosolic compartments, but also within the two DNA-containing organelles, the mitochondria and chloroplasts. Chloroplast genomes of angiosperms code for about 80 proteins. In addition, there are genes for tRNAs and rRNAs; rRNAs are 70S ribosomes that translate all chloroplast mRNAs.

The sub-organellar localization of chloroplast RNAs has rarely been addressed, but there are a few notable exceptions. In the green algae *Chlamydomonas reinhardtii*, several chloroplast mRNAs (e.g., *psbC*, *psbD*, *rbcL*) have been demonstrated to be enriched around the chloroplast pyrenoid [4,5] by fluorescence in situ hybridization (FISH). The pyrenoid seems to be a dominant site of mRNA translation and thylakoid membrane complex assembly in this algae [6]. A large fraction of all Chlamydomonas chloroplast 70S ribosomes is attached to thylakoid membranes, as evidenced both by electron microscopy as well as cell fractionation [7,8,9]. This suggested early on that chloroplast mRNAs are translated in close association with chloroplast membranes. Since many chloroplast mRNAs encode integral membrane proteins, it was assumed that translating ribosomes get trapped on membranes because the nascent protein chain is translated directly into the thylakoid membrane. This is supported by the finding that membrane attachment of RNAs depends on active translation [10] and is proportional to overall protein synthesis within thylakoid membranes [11]. These findings imply that the membrane-bound ribosomes are associated with mRNAs. This is supported by in vitro experiments, where washed thylakoids are capable of synthesizing proteins when supplemented with soluble factors [12,13]. Recently, ribosome-protected mRNA fragments in the soluble fraction of chloroplasts were compared with ribosome-protected mRNA fragments in the membrane fraction. This demonstrated transcriptome-wide association of mRNAs coding for membraneous proteins to chloroplast membranes [14]. Noteworthy, mRNA association via nascent-chain bound ribosomes with membranes is astonishingly stable and occurs even in the absence of chlorophyll production [15].

Chloroplast mRNAs are produced as polycistronic units and are subsequently processed [16]. RNA splicing, endonucleolytic cleavage and exonucleolytic trimming and decay gives rise to complex transcript patterns. For most transcript units, the biological importance of this complexity is unclear. Some processing events have been shown to be required for producing translatable messages (e.g., [17]). In maize chloroplast development, a correlation of the accumulation of monocistronic transcript isoforms with their translational efficiency has been noted [18]. In general, however, both unprocessed polycistronic as well as processed monocistronic forms are supposed to be translated [14,19]. How prevalent differential translation of longer or shorter transcript isoforms is in the chloroplast transcriptome is largely unknown.

We set out to determine which mRNAs are associated with chloroplast membranes versus free mRNAs in the chloroplast stroma fraction. Our results suggests that individual transcripts from a single operon show preference for either stromal or membrane association. In rare cases such associations are subject to developmental change, but in general, membrane-enrichment of plastid RNAs is constant during chloroplast development.

## 2. Results and Discussion

### 2.1. Preparation of Sub-Organellar Fractions Highly Enriched in Membrane and Stroma Marker Proteins

**Figure 1:**
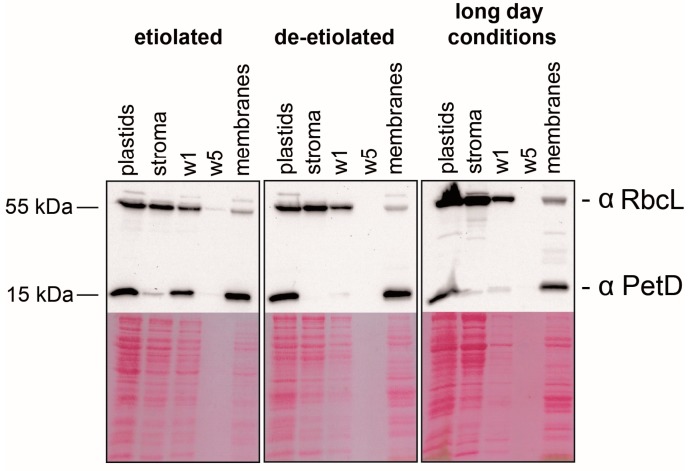
Enrichment of marker proteins in chloroplast membrane and stroma fractions. Western blot analysis was performed from each type of tissue using RbcL and PetD antisera as markers for stroma and membrane, respectively. RNAs from these fractions were used for microarray analysis. A seven hundredth of each sample was analyzed by sodium dodecyl sulfate polyacrylamide gel electrophoresis (SDS-PAGE). The membrane fraction was washed five times prior to RNA extraction. Aliquots from the supernatants of the first and the last wash were analyzed here as well (W1 and W5).

We prepared membranes and stroma extracts from isolated plastids from 9-day-old *Zea mays* seedlings that were either grown in the dark (i.e., are etiolated), from greening seedlings (grown in the dark and then irradiated for 16 h, then kept in the dark for an 8 h night and harvested next morning after an additional 75 min of light exposure), or from plants grown under standard long-day conditions. The three tissue types represent etioplasts, greening plastids, and mature chloroplasts, respectively. The fractions were analyzed immunologically for the presence of the marker proteins PetD and RbcL (Figure 1). PetD is a membrane protein of the cytochrome *b_6_f* complex; RbcL is a stromal protein. There is a slight signal for PetD in stroma from etiolated plants, while the protein is beyond the detection limit in the two other stroma preparations. A small fraction of RbcL can be found within each membrane fraction, indicating a slight contamination of our membrane preparation with stromal proteins. Nevertheless, this immunological analysis shows successful enrichment for both, membrane and stroma proteins, in the two fractions.

### 2.2. Global Association of Chloroplast mRNAs for Membrane Proteins with Chloroplast and Etioplast Membranes

**Figure 2:**
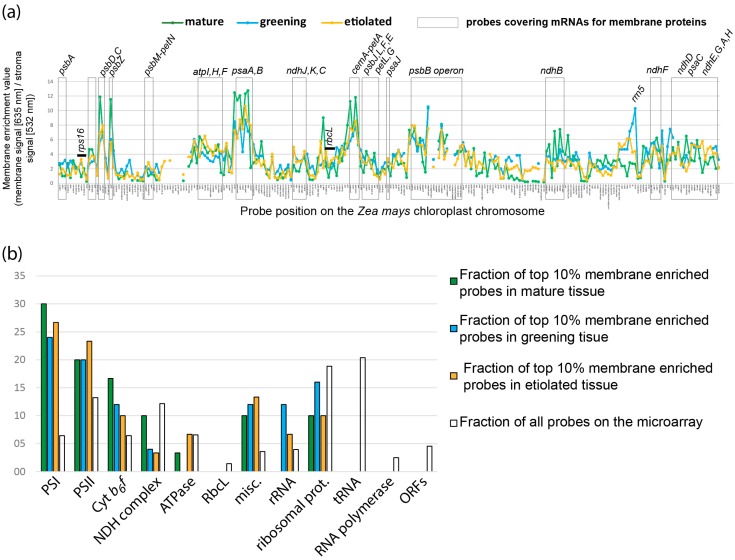
Microarray analysis of RNAs enriched at chloroplast membranes. (**a**) Membrane-enrichment of RNA along the chloroplast chromosome. Isolated chloroplast from maize seedlings grown at three different light regimes (leading to mature, greening, and etiolated plastids, respectively) were processed into stroma and membrane fractions. Two µg of RNA isolated from each fraction were differentially labeled and hybridized to a microarray representing the maize chloroplast genome in a tiling fashion. The ratio of membrane versus stroma signal was plotted against the genomic position on the *Zea mays* chloroplast genome (acc. NC_001666). The graphs shown represent four biological replicates. Normalization between conditions is based on the sum of membrane enrichment values (MEVs) of all probes for each condition. Probes corresponding to mRNAs coding for known membrane proteins are highlighted by dashed boxes. Selected soluble RNAs are labelled as well. The data underlying this chart are deposited in Table S1; (**b**) Gene ontology (GO) term analysis of membrane-enriched RNAs in the chloroplast. The probes showing the top 10 percent MEVs binned into 12 functional categories (PSI = photosystem I; PSII = photosystem II; Prot. = proteins; ORFs = open reading frames of unknown function; misc. = miscellaneous). Probes covering more than one category were counted in each relevant bin. The numbers of probes within each bin are expressed as the fraction of the total number of probes for each condition (in %). This is compared to the distribution of all probes on the array across the bins (open bars). Probes for photosystems I and II are over-represented in the top 10% MEV probes of all three tissue types.

To investigate the association of RNA with membranes on a global level, we labelled RNA from stroma and membrane fractions with fluorescent dyes and competitively hybridized them to a whole-genome tiling array of the maize chloroplast genome [20]. We opted for stroma preparations versus total chloroplast RNA as a control sample, since at least some RNA degradation is expected to occur during membrane/stroma preparation, but is largely absent from total chloroplast RNA preparations. The ratio of membrane-signal over stroma signal (the membrane enrichment value (MEV)) was calculated for all probes. MEVs from four biological replicates were normalized, combined and plotted against the genome position of the probes (Figure 2a). A gene ontology (GO) analysis was carried out for the top 10% probes with the highest MEVs in comparison to all probes on the array (Figure 2b; full data set can be found in Table S1). This revealed that probes representing components of photosystem I, II and the cytochrome *b_6_f* complex are overrepresented in the top 10% MEV probes. Several operons containing genes for these complexes are represented by multiple probes of similar MEVs, for example the *psbB*–*petB* and the *psbD/C* operons, which is further support for the validity of the assay. Membrane-based translation of mRNAs coding for photosynthetic proteins has been demonstrated before by ribosome profiling [14]. By contrast, probes for ribosomal proteins, the NADH dehydrogenase (NDH) complex and the ATP synthase are underrepresented in the top 10% MEV probes. Probes for *rbcL*, tRNAs, the plastid RNA polymerase and uncharacterized open reading frames (ORFs) are not found at all among the top 10% MEV probes. This distribution of MEVs was found for each of the three tissues analyzed, i.e., noteworthy also for etiolated tissue (Figure 2 and Table S2).

While most RNAs within the top 10% membrane-enriched fraction are encoding proteins with photosynthetic functions, there are also interesting exceptions, namely *rps14* and *cemA*. Both are represented by multiple probes within the top 10% MEV probes. *rps14* is the only mRNA coding for a ribosomal protein that shows such high enrichment in membranes (Table S2). It is suggested that the *cemA* gene product is required for the import of inorganic carbon across the envelope membrane, but it was not found to be attached to membranes in recent ribosome profiling analysis [14]. Both *rps14* and *cemA* are part of main peaks in this analysis, represented by multiple probes corresponding to the *psaA/B* and the *cemA*–*petA* operon (Figure 2a). *rps14* is accumulating as part of a tricistronic transcript together with *psaA* and *psaB* and *cemA* is located within an operon adjacent to *petA*. *petA* and *psaA/B* encode core membrane proteins of photosystem I and the *cytochrome b_6_f* complex. Thus, *rps14* and *cemA* are likely drawn to the membrane as part of a larger transcript tethered to the thylakoid membrane via translated *psaA/B* and *petA*, respectively.

### 2.3. Membrane Association of Chloroplast RNAs via Ribosomes

**Figure 3:**
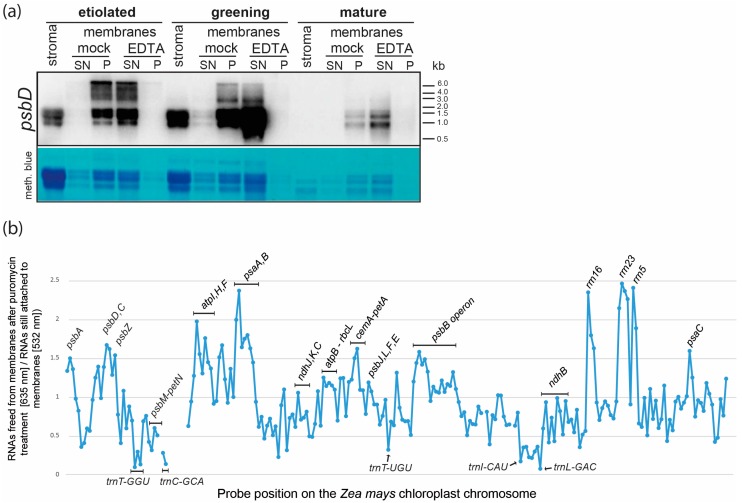
RNAs are associated to chloroplast membranes via translating ribosomes. (**a**) Disruption of ribosomes by ethylenediaminetetraacetic acid (EDTA) releases *psbD* mRNAs into the soluble fraction. Membrane fractions from the same number of plastids from three different tissue types were either treated with EDTA in order to separate the large and small subunit of the ribosome or were treated with buffer (mock). Membranes were spun down after treatment (P) and the supernatant was collected as well (S). RNA was extracted from all fractions and fractionated on 1.2% agarose gel and analyzed by hybridization to a radiolabeled *psbD* probe (see Table S4 for primer sequences). As a quality control, the rRNAs were stained with methylene blue; (**b**) Release of mRNAs from chloroplast membranes after puromycin treatment. Membranes were isolated from mature chloroplasts that were purified from four-week-old plants grown under long-day conditions. Membranes were treated with puromycin to release nascent chains and ribosomes from the mRNAs and thus secondarily free mRNAs from the membrane. The membrane was washed multiple times. RNA from the supernatant after puromycin treatment and from the washed membranes was analyzed by a whole genome tiling array of the maize chloroplast genome. The ratio of freed RNA over membrane-bound RNA was calculated for each probe and plotted against genome position. Selected probes are labelled by the gene-names they represent. The isoaceptor tRNAs are named by their respective codon (GGU, GCA, UGU, CAU and GAC, respectively).

The suggested mechanism of membrane association of chloroplast mRNAs is via actively translating ribosomes [7,9,14]. To test directly for the importance of ribosomes for mRNA-membrane association, we treated membranes with ethylenediaminetetraacetic acid (EDTA) to separate the small and large subunit of the ribosome and thus disrupt membrane and mRNA association. RNA retrieved from supernatants and membranes after EDTA treatment was analyzed by RNA gel blot hybridization using a probe specific for the *psbD* gene (Figure 3a). We observed that in mock-treated samples, the signal for *psbD* mRNAs is almost completely retained in the membrane fractions. Only shorter transcripts of 1.0 to 1.5 kb are found in the stroma of etiolated and greening tissue. These transcripts likely represent monocistronic *psbD* (the *psbD* coding region is 1.0 kb in length) and are in excess of longer precursors. Possibly, such abundant shorter transcripts are not all engaged with ribosomes and can thus partially remain in the stroma. Larger transcripts encompassing *psbD*, *psbC* and further genes are only found in membrane fractions prior to EDTA treatment, which could be explained by their more efficient translation relative to the smaller monocistronic RNAs. After EDTA treatment, no *psbD* remains detectable in membranes and the RNA is instead found in the supernatant. This is further evidence for ribosomes being required for membrane attachment of chloroplast mRNAs. Still, it cannot be ruled out that larger ribonucleoprotein (RNP) granules that would co-enrich with membranes despite a physical interaction, dissolve due to EDTA treatment and are thus released in the supernatant. Such granules have been described in *Chlamydomonas reinhardtii* [21].

Therefore, to validate the hypothesis that nascent chains emerging from ribosomes tether polysomes to chloroplast membranes, we treated mature membranes with puromycin. Puromycin leads to premature peptide chain termination and thus releases ribosomes from mRNAs. We analyzed RNAs released into the supernatant versus membrane-bound RNAs on our microarray after puromycin treatment. As expected, puromycin led to a massive release of ribosomal RNAs from membranes, demonstrating that the antibiotic treatment was successful (Figure 3b). This rRNA release was accompanied by a loss of mRNAs from membranes. mRNAs that showed high MEV values in our previous analysis were particularly prone to detachment from membranes after puromycin treatment. This was for example seen for the *psbD/C* and *psaA/B* operons (Figure 2a and Figure 3b). A similar set of RNAs was found when we treated membranes with EDTA and analyzed the RNAs on microarrays (Figure S1). By contrast, tRNAs were released to a far lesser extent (Figure 3b). tRNAs and also the known stromal mRNAs *rbcL* were found in membranes even after extensive washing. This could be caused by the protection of RNAs within vesicles that are forming from membranes during preparation. In sum, association of mRNAs with membranes was mediated globally by translating ribosomes tethered to membranes via nascent peptide chains.

### 2.4. tRNAs, mRNAs for Stromal Proteins and Short mRNAs for Membrane Proteins Are Enriched in the Soluble Fraction of Plastids

As expected, many tRNAs show low MEVs, in fact the lowest of the entire analysis (Table S2). 79% of all genes represented in the 10% least enriched RNAs across all three tissue types are tRNA genes. An additional 16% of the low MEVs correspond to ORFs of unknown function (Table S2). In order to visualize, which mRNAs show low MEVs, we removed probes for tRNAs and rRNAs from the analysis (Table S3). Expectedly, mRNAs like *rbcL*, *clpP*, *infA* and *matK* that code for soluble proteins do show low MEVs. Similarly, many mRNAs for ribosomal proteins display low MEVs. In addition, few probes for photosynthetic mRNAs coding for membrane proteins have low MEVs as well. Of the latter, almost all represent short reading frames, including *petN* (29 codons), *petL* (31), *petG* (37), *psaI* (36), *psaJ* (42), *psbM* (34) and *psbJ* (40). As has recently been discussed for ribosome profiling, such short mRNAs are fully translated before a sufficiently long peptide chain emerges from the large subunit’s exit channel that would enable contact with the membrane [14]. An exception within this group is *psbA*, which encodes the 40 kDa membrane protein D1 and is 354 codons long. Several probes for *psbA* appear at the bottom of the MEV list for all three tissue types. mRNA for *psbA* is accumulating in large amounts in chloroplasts, mostly untranslated [22,23], and is believed to be required as a reservoir during stress-induced demand for D1 production [24]. Thus, the untranslated excess of *psbA* mRNA likely leads to the observed low membrane enrichment values. In sum, our analysis demonstrates on a transcriptome-wide scale that mRNAs coding for soluble proteins localize to the stroma. In addition, short mRNAs for membrane proteins as well as the *psbA* mRNA are predominantly stroma-localized as well. Paralleling what we observed for membrane-associated RNAs, the list of soluble RNAs is similar between all three tissue types examined.

### 2.5. Etioplasts Display Membrane Enrichment of RNAs Similar to Chloroplasts

Etioplasts differ dramatically in their internal structure from structures known in developing and mature chloroplasts, showing a para-crystalline prolammelar body (PLB) instead of thylakoid membranes. Thus, it came as a surprise that the membrane enrichment curves of plastid RNAs are similar between the three plastid types analyzed (Figure 2a). To uncover potential differences, we did a pairwise comparison of the MEV datasets (Figure S2). This corroborated the large similarities found in the operon (Figure 2a) and GO term analysis (Figure 2b). Intriguingly, greatest similarities are not found between the two green plastid types, but between etioplasts and plastids from greening tissue (Pearson correlation *r*_E/DE_ = 0.83; Figure S2). By contrast, transcript enrichment at membranes in mature chloroplasts are less similar to the two other plastid preparations (*r*_DE/G_ = 0.66; *r*_E/G_ = 0.72, Figure S2). Possibly, the short time span of irradiation has not sufficed to erase the etioplast transcript pattern in greening plastids, thus making these two plastid types more similar on the RNA level and thus also membrane enrichment level than either is to mature chloroplasts. By contrast, the translational activity will have much increased in the greening plastids, but has not led to stronger membrane attachment in our dataset nor in a previous analysis of selected mRNAs [25].

Overall, the most pronounced difference was found for the aforementioned ORFs. The region encompassing ORF46 to ORF137 shows a higher membrane enrichment in etiolated and greening versus mature tissue (Figure S2). The ORFs in question are remnants of a long reading frame of unknown function called *ycf2* [26]. Their low spot count, indicative of a low expression level, suggests that further, more sensitive assays will be necessary to ascertain the differential membrane association seen here.

Why should there be mRNA association with membranes in etioplasts at all? 95% of all prolammelar body (PLB) proteins are protochlorophyllid oxidoreductases (PORA and PORB), which are encoded in the nuclear genome [27,28,29,30,31]. In addition, a number of proteins involved in the light reactions of photosynthesis has been identified. This includes the soluble chloroplast-encoded proteins AtpA, AtpB, RbcL, as well as membranous, chloroplast-encoded proteins like the subunits of the cytochrome b6f complex, and the PSII subunit PsbE [28,30,32,33,34]. Thus, etioplast do have an active translation system. We conclude, that the observed association of many mRNAs coding for membrane proteins with etioplast membranes is reflecting this translational activity (Figure 2 and Figure 3; [14,35]). It remains however unclear, to which membrane type these RNAs are bound, since we did not differentiate between etioplast envelope membranes, the PLB and lamellar prothylakoid membranes present in various cell types of etiolated maize leafs [36]. Also, we cannot exclude that the green light we utilize during etioplast preparation affects translational activity and thus mRNA localization.

In contrast to the examples listed above, many other of the chloroplast-encoded proteins of the thylakoid membrane complexes have not been detected in etioplasts. These appear to be only accumulating upon illumination. For example, the photosynthetic membrane proteins D1, PsbB, PsbC or the PSI reaction core proteins are not or only barely detectable in etioplasts, but have been shown to exhibit a massive increase in expression during photomorphogenesis [25,33,37]. We show here on a genome-wide scale that this increased expression is not reflected by a comparable increase in association of the corresponding mRNAs to chloroplast membranes in greening versus etiolated tissue. Rather, membrane association of plastid mRNAs appears stable across the three plastid types analyzed here. Even *psbA* and *psbB*, which show induction of mRNA levels in greening tissue, exhibit only a mild change in the ratio of membrane-bound versus stroma-bound RNAs. This is in line with previous analyses that show only a slight increase in *psbA* membrane attachment during de-etiolation in barley [38].

### 2.6. Quantitative Analysis of Membrane-Bound and Stromal mRNAs on a “Per-Chloroplast” Base Uncovers Transcript-Autonomous Localization Patterns

**Figure 4:**
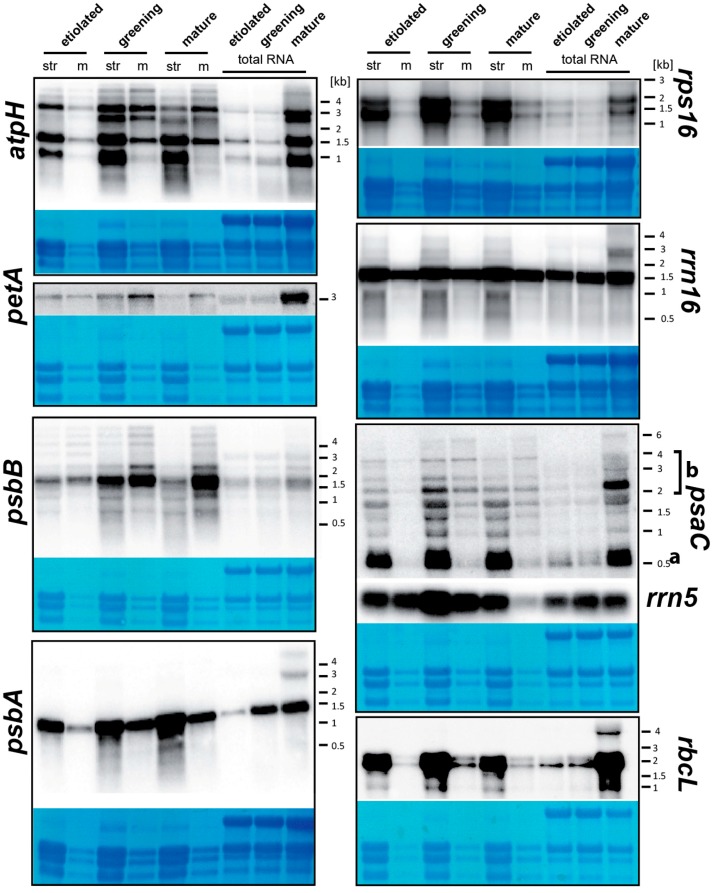
Analysis of transcript accumulation in membrane and stroma preparations on a per-chloroplast base. Equal aliquots of RNA from membrane and stroma fractions of purified plastids from three different tissues were extracted from the same number of chloroplasts. In addition, five micrograms of total leaf RNA was analyzed as well. The RNAs were fractionated on 1.2% agarose gel and analyzed by hybridization to radiolabeled probes for the plastid RNAs indicated (see Table S4 for primer sequences). As a quality control, the rRNAs were stained with methylene blue. Str = RNA from stroma fractions; m = RNA from membrane fractions.

Our array analysis indicates association of RNAs with chloroplast membranes, but the resolution of this approach is limited by the number and length of probes on the microarray. Thus, the microarray analysis misses the great variety and complexity of transcripts representing individual genes within each operon. To understand which RNA species are bound to membranes, we performed quantitative RNA gel blot hybridization assays. We again extracted chloroplasts from the three maize cultures and followed the same procedure for RNA preparation from membrane and stroma fractions as for the microarray approach, with the exception that we counted chloroplasts prior to lysis and cell fractionation. This way, we could normalize the RNA amount from stroma and membrane preparations to chloroplast numbers and thus represent the quantitative differences in RNA abundance between the two fractions more accurately than when total RNA amounts are used for normalization. We utilized probes for eight different chloroplast genes that can be divided into two groups: a group of genes coding for membrane-proteins; and a second group including mRNAs coding for soluble proteins and RNAs that are not translated. *atpH*, *petA*, *psbB*, *psbA* represent the former group, while *psaC*, *rps16*, *rbcL*, *rrn5* and *rrn16* represent the latter. The RNAs we recover from the chloroplast fractions are intact: there are no bands found that would not also be seen in total RNA preparations (Figure 4).

#### 2.6.1. The Analysis of Equal Amounts of Total Cellular RNA from Different Tissues Does Not Accurately Reflect per Organelle RNA Levels for Most Plastid Genes

All RNAs analyzed are found to be expressed in etiolated, greening and mature plastids. In etiolated tissue, there is overall less RNA per plastid than in the two photosynthetic tissues examined (Figure 4). This is most pronounced for RNAs related to the light reactions of photosynthesis (*psaC*, *petA*, *psbB*, *atpH*, *psbA*) and to a lesser extent also for *rbcL* and genes of the genetic apparatus (*rps16*, *rrn5*). 16S rRNA accumulation remains constant across all three conditions. For a number of RNAs, peak accumulation levels are found for greening tissue rather than for mature tissue (e.g., *rrn5*, *rbcL*, *atpH*). Hence, in line with previous analyses, there is a global dependency of chloroplast RNA accumulation on light signals and chloroplast stage. Whether the observed smaller differences in the extent and timing of induction are biologically relevant, is unclear at present.

When considering total RNA preparations (lanes loaded with equal total cellular RNA amounts), there is dramatically less chloroplast RNAs in etiolated and greening tissue than in mature tissue for most genes analyzed. Apparently, the amount of RNA per chloroplast remains constant during initial chloroplast development while, later, the ratio of plastid RNA to cytoplasmic ribosomal RNAs increases towards mature chloroplasts. Again, only the 16S ribosomal RNAs displays an approximately equal accumulation in total RNA across the three conditions. In addition, *psbA* and *rrn5* display induction of RNA levels already in total RNA preparations of greening tissue. Apparently, RNA accumulation is reacting faster to irradiation for these two genes than for the other genes assessed here. A rapid response of *psbA* on all levels of gene expression has indeed been described in various plant species [33,39]. In general, our analysis demonstrates that changes observed for chloroplast-encoded transcripts in different tissues or conditions are pronouncedly dependent on whether normalization is made according to total cellular or total chloroplast RNAs.

#### 2.6.2. mRNAs Are Enriched at Chloroplast Membranes in a Transcript-Autonomous Fashion

The RNA gel blot hybridizations corroborate microarray findings. For example, RNAs for *psbB* and *petA* that display the most pronounced membrane-bias in the northern analysis do also display high MEVs in microarrays. RNAs found enriched in the stroma in RNA gel blots like *rbcL* and *rps16* have comparatively low MEVs (compare these four mRNAs in Figure 2a and Figure 4). In line with this, mRNAs for *atpH*, *psbA*, and the extrinsic membrane proteins *psaC*, display low membrane enrichment values in the microarray analysis and are also found predominantly in stroma fractions in RNA gel blots. This demonstrates a qualitative congruence of the two assays.

For all probes, we noted that the qualitative transcript patterns are similar between stroma and membrane fractions: All transcripts found in the stroma can also be detected in the membrane fraction for any of the probes used (Figure 4). Intriguingly, different transcripts detected with the same probe display distinct distributions between membrane and stroma. A striking example for this is the *psaC* probing, which shows several transcripts highly enriched in the stroma fraction, and in addition larger transcripts that are approximately equally distributed. Most prominent is a band of less than 0.5 kb (labelled “a” in Figure 4), which corresponds to the monocistronic form of *psaC* [40] and is found almost exclusively in the stroma fraction. This RNA species has been described as the major translatable RNA species in tobacco [40,41]. Given that PsaC is translated as a soluble protein and only later assembled into PSI [42,43], it is not surprising that its translated mRNA is found in the stroma. Longer transcripts are however found in membrane fractions (summarized as “b” in Figure 4). These are co-transcripts including cistrons encoding membraneous subunits of the NDH complex (e.g., NdhD). These likely draw the *psaC*-cistron to membranes in the process of their translation. Similarly, monocistronic *atpH* transcripts are found almost exclusively in the stroma, while the long, polycistronic precursors are co-fractioning also considerably with membranes. An additional cistrons in the longer transcripts is represented by *atpF*, which is translated on membranes [14]. Thus, the membrane-attachment of polycistronic *atpH*–*atpF* transcripts can be explained by the affinity of the *atpF* mRNA for membranes. As a general trend, different transcripts from complex operons can show differential association with membranes in chloroplasts.

#### 2.6.3. Differential Membrane-Association of rRNA Species

If a number of chloroplast mRNAs display membrane-association and if this association is mediated by ribosomes, then we should find rRNA attached to chloroplast membranes as well. Indeed, we do observe that 16S rRNA and 5S rRNA co-fractionate with membranes (Figure 4). The unexpected finding is, that different rRNA species show different MEVs. While the ratios of membrane versus stroma signal of 16S and 23S rRNAs remain constant across the three conditions analyzed, 5S rRNA displays a strong decline of membrane association in tissue with mature chloroplasts, which is also reflected by our microarray analysis (see *rrn5* in Figure 2a). 5S rRNA and 23S rRNA are part of the large ribosomal subunit. Thus, the finding that a subpopulation of 5S rRNA localizes to the stroma in mature chloroplasts suggests that it does so independently of the ribosome. Alternatively, a recently discovered antisense RNA to 5S may cause this discrepancy [44]. We cannot distinguish between sense and antisense transcripts with our microarray nor with the double-stranded probe used to detect 5S rRNA on RNA gel blots. Whether the tissue-dependent localization of 5S rRNA is of functional significance remains to be determined.

#### 2.6.4. Constant Association of Plastid RNAs with Chloroplast Membranes Suggests Altered Translational Rates during Chloroplast Greening rather than Increased Accumulation or Improved Recruitment of mRNAs to Membranes

Consistent with the microarray analyses, the RNA gel blot hybridizations also shows a striking similarity in RNA distribution between membranes and stroma in the three tissue types (Figure 4). mRNAs for the soluble proteins *rbcL*, *rps16* and *psaC* display almost identical preferential localization to the stroma fractions with only minor amounts within the membrane preparation in all three tissues. A noticeable, albeit small shift of RNAs towards membrane pools in mature chloroplasts was observed for *psbB* and *petA*, and a minor shift also for *psbA*. *psbA* translation is massively induced after irradiation [33,39], far exceeding the minor increase in membrane attachment seen here. Given that in general, translational induction in the light is known to be massive for many mRNAs [33], we have to assume that RNA tethering to membranes does not directly mirror translational activity. This is reflecting previous analyses in *Chlamydomonas* that found EF-Tu and *psaA/B* mRNAs are strongly and consistently attached to chloroplast membranes throughout dark-light cycles, while translation occurs only in the light [45]. Similarly, the barley *psaA/B* mRNA associates with chloroplast membranes in etioplasts without noticeable *psaA/B* translation [25,38]. It was suggested that there is active repression of *psaA/B* translation in the dark since translation off of isolated etioplast membranes could be initiated in vitro after adding a translation-competent extract [25]. This is supported by studies in pea that report that membrane-bound, non-polysomal RNAs are moving within 8 min into polysomes after illumination, suggesting that the initial association with membranes is either mediated by one or few ribosomes or by other means [10]. Increasing translation from low levels to high levels would not necessarily lead to an increased detection of mRNAs in membranes, since at least in theory, it would not matter for our approach if few or many ribosomes tether an mRNA to thylakoid membranes. Quantitative ribosome profiling experiments in etioplasts versus chloroplasts could solve this problem.

In sum, our analyses demonstrate that etioplasts are poised for a rapid translational answer to light signals on the level of global mRNA attachment to chloroplast membranes. This likely goes hand in hand with parallel processes that stage the necessary production of mRNAs and the corresponding expression factors in etioplasts [30,32]. It remains to be shown, how important constant attachment of mRNAs to membranes is for rapid photomorphogenesis versus other processes, like the provision of RNA binding proteins for translational initiation.

## 3. Materials and Methods

### 3.1. Plant Material

Wild type maize seedlings were grown on soil for 8 days with 16 h light/8 h dark cycle at 25 °C. Etiolated tissue was grown without light for 8 days and harvested directly. Greening tissue was generated by growing plants for 6 days without light and the light-exposure on the seventh day for 16 h and subsequently return into darkness for 8 h. On the 8th day the tissue was exposed for a further hour and 15 min prior to harvest, which was carried out in safety green light. Tissue with mature chloroplasts was generated by growing plants for 8 days under a standard light regime (16 h light/8 h dark). On the 8th day, mature seedlings were exposed for one additional hour to light after which the tissue was harvested. During plastid isolation from etiolated and greening tissue, safety green light was used, so additional exposure to photomorphogenic light was eliminated. All plants were grown at 26 °C.

### 3.2. Extraction of Stroma and Membrane Fractions

Intact plastids from three different tissue types were isolated from 8-day-old seedlings [46] with the following modifications. After Percoll gradient purification, washed plastids were resuspended in resuspension buffer (50 mM HEPES/KOH pH 8, 330 mM sorbitol) by gentle agitation at 4 °C. Dilution of extracted plastids was used for estimation of the number of plastids per microliter microscopically. Plastids from etiolated, deetiolated and green tissue was adjusted to the same number of plastids per microliter. The plastid pellet was resuspended in hypotonic polysomal buffer [19] without detergents, containing heparin, chloramphenicol, cycloheximide, β-mercatoethanol and protease inhibitor cocktail without EDTA. Chloramphenicol stabilizes ribosomes on mRNAs. Lysis of plastids was performed by passing the extract for 40 to 50 times through a 0.5 mm × 25 mm syringe in a hypotonic buffer designed to keep polysomes intact [19,47]. Membranes and stroma were separated by centrifugation at 40,000× *g* at 4 °C for 30 min. The membrane pellets were washed five to six times in polysomal buffer, and finally resuspended in the same volume as the stroma volume obtained. From equal volume proportions, RNA was isolated. For RNA gel blot analyses, RNAs were loaded on volume basis.

### 3.3. EDTA and Puromycin Treatments of Purified Plastid Membranes

Plastid membrane fractions from all three tissue types were obtained from 8-day-old seedlings. Aliquots (300 μL) were incubated at room temperature with 100 mM EDTA (final concentration) for 15 min with gentle shaking. Control reactions were performed without addition of EDTA. After incubation, RNAs released from membrane particles, were separated by centrifugation for 10 min at 21,000× *g* at 4 °C. Membrane pellets were resuspended in lysis buffer in same volume as the one of the corresponding supernatant. From each of the fractions RNA was extracted by phenol/chloroform/isoamylalcohol (24:24:1).

For puromycin treatment, membrane fractions were incubated in 500 ng/μL Puromycin in polysome buffer [19] for 15 min at room temperature. Afterwards, membranes were pelleted by centrifugation at 21,000× *g* for 10 min at 4 °C. The membrane pellet was washed five times in polysome buffer. RNA was extracted from supernatant (freed RNA) and pellet (membrane-bound RNA) fractions. Two μg of RNA from each fraction were labelled and subjected to microarray analysis see below.

### 3.4. RNA Gel Blot Analyses

Total RNA was extracted from etiolated, deetiolated and green tissue with TRIzol reagent (Invitrogen, Carlsbad, CA, USA) according to the manufacturer’s instructions. For each individual sample a pool of collected leaves was used. Five micrograms of total RNAs from tissue or RNAs extracted from stroma and membrane fractions were separated on an agarose gel containing 1.2% formaldehyde and transferred to uncharged nylon membranes (Hybond N, GE Healthcare, Little Chalfont, UK). After transfer, the blots were UV cross-linked (150 mJ/cm^2^) and then stained with methylene blue to check RNA integrity and loading.

Polymerase chain reaction (PCR) based probes were used for in vitro transcription via T7 polymerase (Thermo Fisher, Waltham, MA, USA) in the presence of 32P-UTP according to the manufacturer’s instructions. Primers used are listed in Table S4. For all northern blots, except *rrn16* and *rrn5*, single stranded in vitro transcripts were used for hybridization. The *rrn16* and *rrn5* RNA gel blots were hybridized with a double-stranded PCR probe labelled in presence of 32P-CTP via a Klenow exo- fragment (Thermo Fisher, Waltham, MA, USA) according to the manufacturer’s instructions. After pre-incubation in Church buffer (0.5 M sodium phosphate buffer, pH 7.2 and 7% sodium dodecyl sulfate (SDS) for 1 h at 68 °C, hybridization of radiolabelled probe was performed overnight at 68 °C in the same buffer, followed by at least three 15-min washes in 1× SSC; 0.1% SDS, 0.5× SSC/0.1% SDS and 0.2× SSC/0.1% SDS, respectively. Ribosomal RNA related probes, *psbA*, and *rbcL* probes were washed additionally for 15 min at 68 °C with 0.1× SSC/0.1% SDS and 0.05× SSC/0.1% SDS. Signals were detected by autoradiography with the Personal Molecular Imager system (Bio-Rad, Hercules, CA, USA).

### 3.5. Immunoblot Analyses

Proteins from stroma and membrane fractions were loaded on a volume-per-volume basis. Proteins were separated by sodium dodecyl sulfate polyacrylamide gel electrophoresis (SDS-PAGE) and transferred to Hybond-C Extra Nitrocellulose membranes (GE healthcare). Integrity and loading of protein samples was detected by Ponceau S stain of the membrane prior incubation with antibodies. Antibody hybridization was performed for 1 h in 2% skim milk powder in TBST buffer for primary antibody and secondary horseradish peroxidase coupled antibody in TBST.

### 3.6. Tiling Microarray Design

Overlapping PCR products covering the whole maize chloroplast genome were generated by using self-made Taq-polymerase and were purified via the QIAquick PCR purification kit (Qiagen, Hilden, Germany). A total of 500 ng of each PCR product was transferred into a 384-well plate, dried and resuspended in 5 μL of 1M betaine in 3× SSC buffer. DNA fragments were transferred on to silanated glass slides (Vantage Silanated Amine Slides; CEL Associates, Los Angeles, CA, USA) using an OmniGrid Accent microarrayer (GeneMachines, San Francisco, CA, USA). Array design as described [20].

### 3.7. Microarray Hybridisation

Microarrays were cross-linked at 250 mJ/cm^2^ in a UV cross linker (GS Gene Linker, Bio-Rad), and blocked with BSA buffer (1% BSA, 0.1% SDS and 5× SSC) for 1 h at 42 °C. The labelled RNAs from stroma and membrane fractions (approximately 15 μL) were mixed, loaded on to array and covered with a cover slip. Hybridisation was performed overnight in a 42 °C water bath in Corning microarray hybridization chambers. Unspecifically bound RNA was washed off the slide by incubation in 1× SSC, 0.2× SSC, and 0.05× SSC for 8 min each on a horizontal shaker at 180 rpm. Slides were dried by centrifugation at 1500 rpm in plate centrifuge and scanned using the ScanArrayGx Plus microarray scanner (Perkin Elmer, Shelton, CT, USA).

### 3.8. Microarray Analysis

Data from four replicate experiments (this totals 48 spots per probe since we were using 12 replicate spots per probe per array) were filtered against elements with low signal-to-noise ratios, and local background was calculated according to default parameters in Genepix Pro 7.0 software (Molecular Devices, Sunnyvale, CA, USA). Only spots with a signal-to-background ratio >4 and for which 60% of pixels have a F532 fluorescent signal >2 SD above background were chosen for further analysis. Fragments for which fewer than half the number of all spots (i.e., less than 24 spots) per array passed these cutoffs were not used for subsequent analyses and appear as gaps when enrichment ratios are plotted according to chromosomal position. Background-subtracted data were used to calculate the median (median of ratios (membrane RNA = 635 nm: stroma RNA = 532 nm)) as described in [21]. This value is called the membrane enrichment value (MEV). Normalization for Figure 2a and Table S1 was done according to the median (median of ratios (F635/F532)) value for all probes with signal above background on each array.

## Supplementary Materials

Supplementary materials can be found at www.mdpi.com/1422-0067/18/9/1881/s1.

## Abbreviations

MEV	Membrane Enrichment Value
NDH	NADH Dehydrogenase
