# Supplementary material for: Stable Membrane-Association of mRNAs in Etiolated, Greening and Mature Plastids
Source: Int J Mol Sci. 2017 Aug 31;18(9):1881. doi: 10.3390/ijms18091881 (PMC5618530; doi:10.3390/ijms18091881)
Supplement: Supplementary file 1 [file ijms-18-01881-s001.zip › Figure S1.pdf]

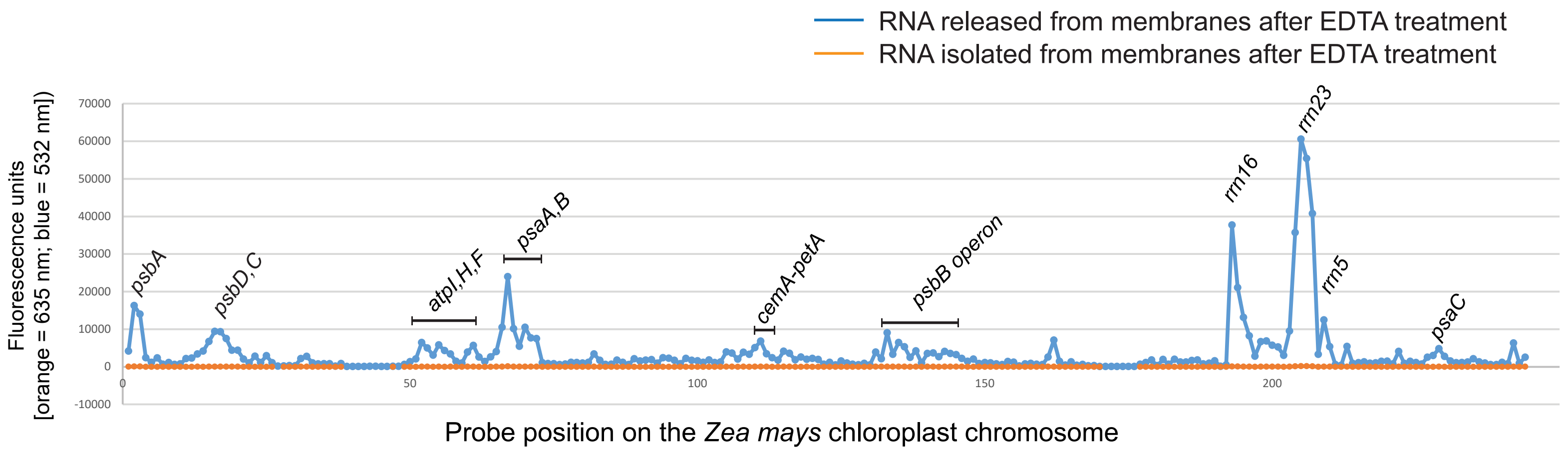

Figure S1: Release of mRNAs from chloroplast membranes after EDTA treatment. Membranes were isolated from mature chloroplasts that were purified from four-week-old plants grown under long-day conditions. Membranes were treated with EDTA to disrupt the small and large subunit of the ribosome and thus secondarily, free mRNAs from the membrane. The membrane was washed multiple times. RNA from the supernatant after EDTA treatment and from the washed membranes was analyzed by a whole genome tiling array of the maize chloroplast genome. The EDTA-released RNA was labelled with Cy3 (detection at 532 nm), while RNA remaining on the membrane was labelled with Cy5 (635 nm). The fluorescence results were background-corrected and plotted against the genome position of the respective probes. Since for many probes, the fluorescence signal was below background levels (negative values), we could not perform a ratio analysis like in Figure 3B. Selected probes are labelled by the gene-names they represent – these probes denote RNAs also found to be released after puromycin treatment.
