# Supplementary material for: Stable Membrane-Association of mRNAs in Etiolated, Greening and Mature Plastids
Source: Int J Mol Sci. 2017 Aug 31;18(9):1881. doi: 10.3390/ijms18091881 (PMC5618530; doi:10.3390/ijms18091881)
Supplement: Supplementary file 1 [file ijms-18-01881-s001.zip › Figure S2.pdf]

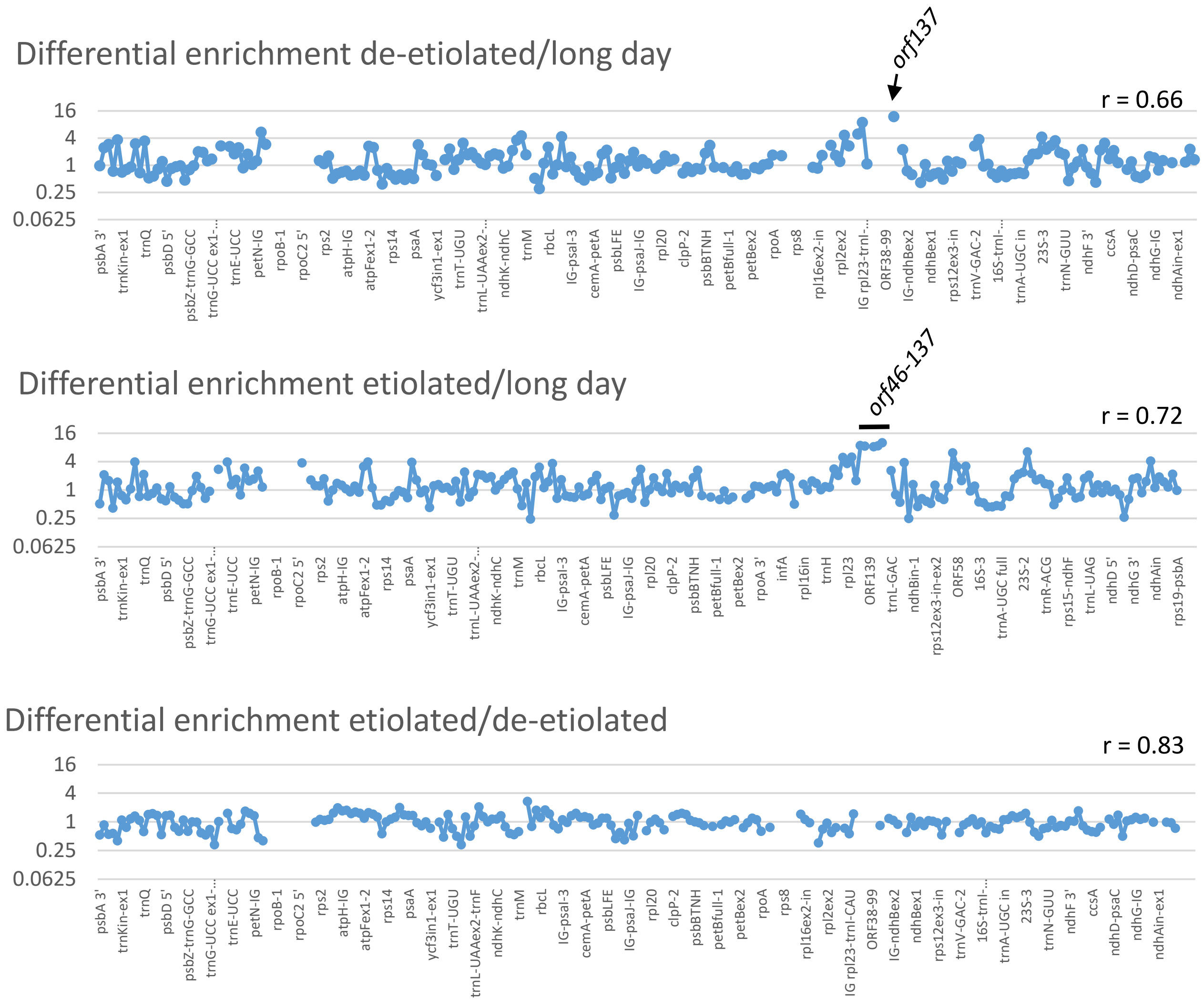

Figure S2: Comparison of membrane enrichment values (MEVs) between different plastid types.

To determine similarities and differences between MEVs in the three plastid types, ratios of MEVs were calculated for all probes on the array and plotted against genomic position. The biggest individual difference is found for ORFs of unknown function that show stronger membrane enrichment in de-etiolated and etiolated plastids versus mature, long-day grown plastids. However, the probes for the ORFs barely passed our quality tests with only marginally more than half of all probes making it into the final analysis (Table S1). As another measure of similarity, we performed a Pearson correlation analysis that further supports the overall high similarity between the different data sets (see r-value above each graph). Note that not all probe names can be shown on the X-axis due to space restrictions.
