# Supplementary material for: Stable Membrane-Association of mRNAs in Etiolated, Greening and Mature Plastids
Source: Int J Mol Sci. 2017 Aug 31;18(9):1881. doi: 10.3390/ijms18091881 (PMC5618530; doi:10.3390/ijms18091881)
Supplement: Supplementary file 1 [file ijms-18-01881-s001.zip › Table S1.pdf]

Table S1: microarray data for mRNAs extracted from chloroplast membrane and stroma fractions from three different tissues

				mature (long-day conditions)				greening					etiolated				
probe name	position on array	start of probe[1]	end of probe[1]	spot count[2]	Median (F635 Median - B635)[3]	Median (F532 Median - B532)[3]	Membrane enrichment value[4]	spot count[2]	Median (F635 Median - B635)[3]	Median (F532 Median - B532)[3]	Median (Median of Ratios (635/532))[4]	Membrane enrichment value[4]	spot count[2]	Median (F635 Median - B635)[3]	Median (F532 Median - B532)[3]	Median (Median of Ratios (635/532))[4]	Membrane enrichment value[4]
psbA 3'	1	36	336	48	4848.5	6626.5	2.528	42	3069.5	7437	0.6235	2.43165	48	1748.5	4023.5	0.2495	1.27245
psbA	2	296	1074	48	17691	15278.5	0.988	36	13168	16763	0.6125	2.38875	48	13599	27718.5	0.4	2.04
psbA 5'	3	973	1421	47	7281	4402	0.987	42	12778.5	17856.5	0.7255	2.82945	48	7407.5	27635	0.3025	1.54275
trnKex2-in	4	1261	1710	48	3485	2085	2.739	35	5220	11860	0.507	1.9773	43	3972	17712	0.218	1.1118
matK	5	1677	3308	24	1526.5	2250.5	0.756	32	2142.5	1417	0.7	2.73	26	967	5214	0.2135	1.08885
trnKin-ex1	6	3185	4197	48	2663.5	2378	3.1135	36	2154	1960	0.5545	2.16255	48	2532	4922	0.4585	2.33835
IG-rps16 ex2	7	4085	4712	48	1327	800	2.2465	42	495.5	2012.5	0.4615	1.79985	48	1487.5	3783.5	0.2695	1.37445
rps16 full	8	4521	5916	48	2163	1997.5	2.146	42	1485	2754.5	0.495	1.9305	48	1362.5	3977	0.438	2.2338
rps16 in	9	4751	5561	45	1359	1916	0.87	27	1265	1116	0.66	2.574	45	880	3227	0.66	3.366
IG rps16-trnQ	10	5541	6816	48	1402.5	2518	2.0295	42	753.5	2176	0.348	1.3572	48	2272	4888.5	0.2845	1.45095
trnQ	11	6724	6966	48	1458	6007	0.252	39	5440	31509	0.219	0.8541	48	4439	20701	0.104	0.5304
IG-psbK	12	6929	7380	48	4072	989	4.6595	42	1391	3018	0.6135	2.39265	48	4009	5702	0.66	3.366
psbK-psbI	13	7177	7881	48	5922	4227	4.646	41	4249	4839	0.674	2.6286	48	4573	6763.5	0.76	3.876
psbI	14	7709	7976	48	2042	1223	3.3535	45	1427	1587	0.695	2.7105	48	2465	4187	0.7195	3.66945
trnS-GCU	15	7942	8842	48	3852.5	3454.5	1.6765	42	11231	16580.5	0.5125	1.99875	48	4453.5	12017.5	0.209	1.0659
psbD 5'	16	8739	9862	47	9091	1476	11.89	39	12515	6027	1.33	5.187	48	5115.5	5875	1.3595	6.93345
psbD-psbC	17	9759	10826	48	9924.5	1253	7.0435	42	6677	3630.5	1.502	5.8578	48	4189	1585.5	1.584	8.0784
psbC-trnS	18	10694	11758	48	6183	3038.5	3.3575	42	3906.5	4005.5	0.794	3.0966	48	2430.5	3359	0.459	2.3409
trnS-UGA	19	11661	12000	48	2771.5	6778	1.1455	42	3203	11825	0.2805	1.09395	48	2072.5	8482.5	0.134	0.6834
psbZ	20	11896	12411	48	4546	1091	11.53	33	6993	1144	1.37	5.343	48	2219.5	1617.5	1.131	5.7681
psbZ-trnG-GCC	21	11980	12883	48	789	207	4.488	31	2758	1057	0.91	3.549	35	543	804	0.439	2.2389
trnG-GCC	22	12431	12626	47	1250	1127	1.317	40	700.5	2505	0.3255	1.26945	40	1674.5	6910	0.2465	1.25715
trnfM	23	12762	13305	48	3234	10242	0.5845	42	5979.5	21910.5	0.2935	1.14465	48	3400	17636.5	0.22	1.122
trnfM-trnG-UCCex1	24	13050	13239	48	964	3612	0.9055	45	1557	4269	0.442	1.7238	48	1431.5	6530.5	0.1985	1.01235
trnG-UCC full	25	13248	14013	48	3747	8512	0.987	42	3539.5	16622.5	0.312	1.2168	48	2242	9363	0.126	0.6426

				mature (long-day conditions)				greening					etiolated				
probe name	position on array	start of probe[1]	end of probe[1]	spot count[2]	Median (F635 Median - B635)[3]	Median (F532 Median - B532)[3]	Membrane enrichment value[4]	spot count[2]	Median (F635 Median - B635)[3]	Median (F532 Median - B532)[3]	Median (Median of Ratios (635/532))[4]	Membrane enrichment value[4]	spot count[2]	Median (F635 Median - B635)[3]	Median (F532 Median - B532)[3]	Median (Median of Ratios (635/532))[4]	Membrane enrichment value[4]
trnG-UCC ex1-ORF69	26	13895	14972	48	1220.5	2765	1.5555	42	1452.5	2454.5	0.537	2.0943	48	2083	3582	0.2835	1.44585
ORF69-IG	27	14561	15500	20	654	1623.5		24	1826.5	2079.5	0.723	2.8197	24	385.5	1497	0.1825	0.93075
IG-trnT-GGU	28	14860	16001	39	784	5829	0.394	41	703	2517	0.268	1.0452	48	665.5	2335	0.208	1.0608
trnT-GGU	29	15885	16001	14	1417	3464.5		22	1500	2507			24	411.5	8558	0.1265	0.64515
IG-trnT-GGU-IG	30	15885	16487	39	618	2077	0.379	26	473	2484.5	0.251	0.9789	44	413.5	2478	0.288	1.4688
trnE-UCC	31	16391	17004	48	1575	16491.5	0.3885	42	3688	23395	0.175	0.6825	47	3710	20759	0.096	0.4896
trnE-UCC-trnY-GUA	32	16391	16580	48	2011.5	17838.5	0.3155	36	5314	34491.5	0.196	0.7644	48	3209.5	20829	0.102	0.5202
psbM-IG	33	18178	18681	38	1654.5	1296.5	2.246	30	2091.5	3661	0.4995	1.94805	38	1515.5	4521	0.3375	1.72125
IG psbM-petN	34	18541	19090	48	861	1800.5	1.016	36	986	1935	0.4515	1.76085	48	1266	4013.5	0.573	2.9223
petN	35	19081	19186	48	2421.5	1985	1.2615	42	1351	5052	0.332	1.2948	48	1272.5	1575	0.381	1.9431
petN-IG	36	19081	19396	48	1443	3068	1.133	37	762	2385	0.36	1.404	47	1341	2302	0.37	1.887
IG-trnC-IG	37	19381	20368	29	261	1095	0.435	25	342	1015	0.595	2.3205	27	158	1150	0.211	1.0761
trnC IG	38	20140	20368	48	768.5	4969.5	0.4545	38	1879.5	8494.5	0.333	1.2987	48	1773.5	10756.5	0.1015	0.51765
IG trnC-rpoB	39	20221	21475	23	322	1096		19	1376	3038			25	496	2787	0.454	2.3154
rpoB 5'	40	21380	22732	23	566	1452		20	715	1175			28	326	1054	0.609	3.1059
rpoB-1	41	22638	23370	20	611	1443		20	1306	3646			19	140	944		
rpoB-2	42	23258	24475	23	782	1733		21	415	1623			32	741.5	1227.5	0.6245	3.18495
rpoB-rpoC1	43	24377	25937	14	325.5	795		14	690.5	1458			20	59.5	358.5		
rpoC1 3'	44	25831	27000	21	355	1006		18	695	1945			22	143	901		
rpoC1-rpoC2	45	26739	27040	18	396	1129		19	2071	3534			20	179.5	991		
rpoC2 5'	46	26906	28192	20	399.5	1005.5		20	1266	2715.5			22	118	697		
rpoC2-1	47	28094	29747	24	546	1973	0.3325	21	2191	4748			26	194.5	1126.5	0.239	1.2189
rpoC2-2	48	29641	31009	19	525	1148		21	868	1988			22	185	1045		
rpoC2-rps2	49	30904	32066	48	1156	628	2.3105	22	5170.5	4823.5			38	1352	3687.5	0.724	3.6924
IG-rps2	50	31541	32484	48	1809.5	632.5	3.036	32	2247.5	3228	0.9715	3.78885	40	2120	3614.5	0.7285	3.71535
rps2	51	31951	32697	48	2170	695.5	4.9455	30	5099	1471.5	1.3705	5.34495	44	1902	2329.5	1.174	5.9874

				mature (long-day conditions)				greening					etiolated				
probe name	position on array	start of probe[1]	end of probe[1]	spot count[2]	Median (F635 Median - B635)[3]	Median (F532 Median - B532)[3]	Membrane enrichment value[4]	spot count[2]	Median (F635 Median - B635)[3]	Median (F532 Median - B532)[3]	Median (Median of Ratios (635/532))[4]	Membrane enrichment value[4]	spot count[2]	Median (F635 Median - B635)[3]	Median (F532 Median - B532)[3]	Median (Median of Ratios (635/532))[4]	Membrane enrichment value[4]
IG-atpI	52	32585	32930	37	5746	7049	2.778	42	10148.5	5941.5	1.1355	4.42845	37	4266	13077	0.929	4.7379
atpI-1	53	32820	33563	48	5553.5	3062.5	6.412	42	2680	3287.5	0.8385	3.27015	48	5374.5	8076.5	0.72	3.672
atpI-2	54	32820	33563	48	5617	1131.5	5.861	42	1793.5	2091	0.9615	3.74985	48	2429.5	3114	1.1225	5.72475
IG-atpH	55	34031	34610	48	7760.5	4117.5	4.8825	42	4776	7927.5	0.8675	3.38325	48	5470	1883	1.299	6.6249
atpH-IG	56	34361	35021	48	6630	2212.5	4.1625	42	3051.5	3621.5	0.787	3.0693	48	4425.5	6719.5	1.0135	5.16885
atpH-atpFex1-in	57	34473	35705	48	5725	4041.5	4.56	42	2393.5	5539.5	0.6985	2.72415	48	3125	5537.5	0.925	4.7175
atpFex1-1	58	34941	35385	48	3109.5	1377	4.2875	31	1556	3256	0.671	2.6169	48	1300	2503.5	0.7585	3.86835
atpFin	59	34941	35385	48	1164	783	4.49	28	2017.5	911	0.848	3.3072	39	907	2121	1.041	5.3091
atpFin-ex2	60	35232	35815	48	4530.5	2526	5.3175	42	3586	2689.5	0.812	3.1668	48	3777	1485.5	0.9265	4.72515
atpFex1-2	61	35706	36593	48	7966.5	3362	1.4465	41	7035	8346	0.965	3.7635	48	6614.5	6619	0.8705	4.43955
atpA-5'	62	36590	37680	48	2097.5	918	1.1445	42	1214	1100	0.724	2.8236	48	2184	3883	0.8635	4.40385
atpA	63	37398	37921	48	1761.5	277.5	5.112	31	2268	829	1	3.9	43	1243	2169	1.106	5.6406
atpA-3'	64	37781	38301	48	3595	3197	3.7275	42	1335	7670.5	0.3595	1.40205	48	3088	6651.5	0.3445	1.75695
trnR-rps14	65	38106	39060	48	4440	2946.5	3.0395	42	5580	7056.5	0.656	2.5584	48	2086.5	6792	0.2795	1.42545
rps14	66	38500	39020	48	17247	5941.5	12.4585	41	18351	7475	1.924	7.5036	48	17205	10917.5	1.4515	7.40265
rps14-psaB	67	38929	39260	48	24395	10869.5	11.4355	42	30300.5	12334.5	1.427	5.5653	48	11962.5	10893	1.2385	6.31635
psaB-1	68	39935	41083	48	7478.5	607.5	12.043	42	5111.5	2627	1.848	7.2072	48	5506	4127	1.749	8.9199
psaB-2	69	40721	41090	48	5593.5	819	8.0185	42	3744.5	2840	0.9795	3.82005	48	6489.5	1192.5	1.491	7.6041
psaB-psaA	70	40978	42044	48	13052.5	1980	12.3	42	9618.5	3127	1.9815	7.72785	48	9285	6710.5	2.1255	10.84005
psaA	71	41919	42984	48	10018	2498.5	12.74	42	5036	3115.5	1.622	6.3258	48	6624	7148.5	1.69	8.619
psaA-3'	72	42883	43907	48	8093	2109	2.126	42	6945	2133.5	1.527	5.9553	48	5157.5	3836	1.587	8.0937
IG-ycf3ex3-in2	73	43801	44873	48	1132	256.5	2.7945	24	4148.5	2582	1.215	4.7385	44	1029	1388.5	0.879	4.4829
ycf3in2	74	44290	45121	48	1853.5	682.5	3.251	39	764	1220	0.871	3.3969	48	1309	2405	0.547	2.7897
ycf3ex2-in1	75	45010	46084	48	1669.5	609	3.28	35	1838	1348	0.83	3.237	48	1650.5	2935	0.6345	3.23595
ycf3in1-ex1	76	45820	46220	38	2834	463	5.744	30	1174.5	2379	0.854	3.3306	35	2450	8755	0.475	2.4225
ycf3in1-ex1-IG	77	45982	46520	48	861.5	637.5	2.606	22	11341	11578.5			47	865	3655	0.616	3.1416
trnS-UCC-rps4-1	78	46461	47337	48	977.5	2408	1.281	42	778.5	2283	0.43	1.677	48	1969.5	3731	0.3235	1.64985

				mature (long-day conditions)				greening					etiolated				
probe name	position on array	start of probe[1]	end of probe[1]	spot count[2]	Median (F635 Median - B635)[3]	Median (F532 Median - B532)[3]	Membrane enrichment value[4]	spot count[2]	Median (F635 Median - B635)[3]	Median (F532 Median - B532)[3]	Median (Median of Ratios (635/532))[4]	Membrane enrichment value[4]	spot count[2]	Median (F635 Median - B635)[3]	Median (F532 Median - B532)[3]	Median (Median of Ratios (635/532))[4]	Membrane enrichment value[4]
trnS-UCC-rps4-2	79	46700	47229	42	686	4020	0.723	42	859	3432.5	0.421	1.6419	47	1888	5774	0.152	0.7752
rps4-IG	80	47230	48071	48	1571	1020.5	3.6635	41	842	1514	0.745	2.9055	48	1453	2714.5	0.8035	4.09785
trnT-UGU	81	47975	48660	48	672	2274	0.853	42	842.5	2524	0.2875	1.12125	47	1478	1904	0.157	0.8007
trnT-UGU-IG	82	48066	48215	47	1783	3694	0.477	42	5990	20840.5	0.373	1.4547	48	6419.5	10962.5	0.142	0.7242
IG-trnL-UAAex1	83	48481	49020	48	2261.5	7291	1.301	42	7323	15965	0.5625	2.19375	48	4268.5	9981	0.14	0.714
trnL-UAAex1-in	84	48890	49471	42	437.5	3999.5	0.6905	42	528	1687	0.333	1.2987	48	1156.5	3691.5	0.3185	1.62435
trnL-UAAin-ex2-IG	85	49339	49720	48	1752	4041	1.185	40	2193.5	4995	0.431	1.6809	48	1269	4659.5	0.1625	0.82875
trnL-UAAex2-trnF	86	49471	50041	48	2259	3026	2.034	42	1584.5	3895.5	0.579	2.2581	48	1821	3291.5	0.3625	1.84875
trnF	87	49872	49945	48	908	2171	0.439	40	1186	7465	0.115	0.4485	48	1222.5	7896.5	0.18	0.918
trnF-ndhJ	88	50021	50610	48	907.5	358	2.4975	30	1912	889	1.022	3.9858	44	678	1323	0.996	5.0796
ndhJ	89	50461	51020	48	3121	770	1.674	39	1206	1507	0.762	2.9718	48	2735.5	5585.5	0.578	2.9478
ndhJ-ndhK	90	50981	51360	46	3180.5	1594.5	1.627	42	741	1914.5	0.693	2.7027	47	1627	6566	0.605	3.0855
ndhK-ndhC	91	51275	51931	48	1350.5	533.5	3.128	41	1003	1883	0.686	2.6754	48	1668.5	2931	0.5975	3.04725
ndhC-IG	92	51796	52859	48	1939	892.5	3.5445	37	1219	1288	0.86	3.354	48	1072	1815	0.882	4.4982
IG-trnVex2-in	93	52711	53491	48	756.5	895.5	1.7045	26	1444	911	0.916	3.5724	42	473	1629.5	0.5525	2.81775
trnVex2-in-ex1	94	53158	53834	42	6309.5	12281	0.51	42	3211	7151.5	0.4675	1.82325	48	3052	9243.5	0.2035	1.03785
trnVin-ex1-IG	95	53561	53990	47	4036	11309	0.469	42	4387	6047.5	0.533	2.0787	48	2862.5	8801	0.216	1.1016
trnM	96	53838	54202	48	2250	12346	0.6705	41	3632	27757	0.288	1.1232	48	2877	11469	0.1365	0.69615
trnM-atpE-atpB	97	54098	54690	48	1174.5	273.5	3.4675	20	3098	4895.5			26	350.5	1648	0.3115	1.58865
atpB	98	54591	55790	48	3715	1353.5	4.335	39	2211	2166	0.565	2.2035	47	1184	2350	1.154	5.8854
atpB 5'	99	55692	56410	36	5766.5	672.5	9.0125	29	2327	3156	0.688	2.6832	36	2144.5	4928.5	0.4195	2.13945
IG atpB-rbcL	100	56115	56880	48	2615	1773	2.3385	42	778	1785.5	0.6525	2.54475	48	2557	3097.5	0.8675	4.42425
rbcL	101	56671	57124	48	3482.5	2299	0.7995	40	1233.5	1977.5	0.5085	1.98315	48	3032.5	6586	0.4725	2.40975
rbcL 5'	102	57038	57606	48	5107.5	2904	2.888	42	1233.5	6844	0.466	1.8174	48	4435	8167	0.6275	3.20025

				mature (long-day conditions)				greening					etiolated				
probe name	position on array	start of probe[1]	end of probe[1]	spot count[2]	Median (F635 Median - B635)[3]	Median (F532 Median - B532)[3]	Membrane enrichment value[4]	spot count[2]	Median (F635 Median - B635)[3]	Median (F532 Median - B532)[3]	Median (Median of Ratios (635/532))[4]	Membrane enrichment value[4]	spot count[2]	Median (F635 Median - B635)[3]	Median (F532 Median - B532)[3]	Median (Median of Ratios (635/532))[4]	Membrane enrichment value[4]
rbcL 3'	103	57551	58460	48	1410	835.5	2.3715	37	810	1756	0.617	2.4063	46	821	1627	0.686	3.4986
IG-psaI-1	104	58371	59274	24	1368.5	1343.5	1.0275	30	3029	634.5	1.1155	4.35045	28	841	2841	0.7245	3.69495
IG-psaI-2	105	58496	59506	48	4877	2000	4.375	42	3535	3922.5	1.0315	4.02285	47	3395	6595	0.559	2.8509
IG-psaI-3	106	58496	59506	42	4391	2593.5	3.0485	42	3150.5	2923	1.1655	4.54545	48	4748.5	4256	0.976	4.9776
psaI	107	59151	59301	48	4143.5	1116.5	5.427	42	2320.5	2303.5	1.05	4.095	41	3675	3898	0.78	3.978
ycf4	108	59401	60190	48	4324.5	1907.5	7.985	42	4665.5	1900	1.094	4.2666	48	3681	2957.5	1.1125	5.67375
ycf4-cemA	109	60011	60660	48	2936	1284	11.2605	35	6956	1130	1.326	5.1714	48	3632.5	1900.5	1.5145	7.72395
cemA	110	60470	61484	48	10290.5	1859	6.4655	42	6531	3989	1.5275	5.95725	48	5289	2955.5	1.439	7.3389
cemA-petA	111	61221	61560	48	7843.5	879	11.801	42	3923	2846	1.767	6.8913	48	5626	1025.5	1.716	8.7516
petA	112	61469	62333	48	4036.5	880	6.913	41	2381	2209	1.197	4.6683	48	2480.5	1516.5	1.0985	5.60235
petA 3'	113	62253	63220	47	3496	3560	2.322	42	1462	1308.5	1.036	4.0404	48	2098	2437	0.677	3.4527
psbJ	114	62880	63520	48	2054	926	1.482	36	2224.5	1139	0.8075	3.14925	48	1986.5	4254	0.5785	2.95035
psbJLFE	115	63220	64125	47	5399	2625	4.404	42	3494	6677	0.5845	2.27955	48	4796.5	9333	0.5335	2.72085
psbLFE	116	63541	64320	48	3804	2175	3.3345	41	2120	2966	0.764	2.9796	48	3917	8168.5	0.6945	3.54195
IG psbE-petL	117	64026	65280	48	2428.5	1605.5	2.04	42	1599.5	3249	0.7255	2.82945	47	2289	6955	0.466	2.3766
petL	118	65181	65586	37	6524	2234	4.145	31	8668	15791	0.69	2.691	37	7148	29872	0.234	1.1934
petG	119	65467	65780	48	1727.5	3373	1.3605	42	2501.5	10737	0.4335	1.69065	48	3906	7824	0.1975	1.00725
trnW-trnP	120	65732	66399	48	1580	7769.5	0.659	42	2850	15617.5	0.323	1.2597	48	2075	15909	0.103	0.5253
IG-psaJ-IG	121	66361	67020	48	2843.5	3622.5	2.074	42	1653	2775.5	0.5025	1.95975	48	3174.5	2735.5	0.3525	1.79775
psaJ	122	66399	66673	48	709	503.5	1.925	28	1239.5	1180	0.6465	2.52135	37	2335	19055	0.248	1.2648
psaJ-rpl33	123	66551	67121	48	1578.5	1465.5	2.3525	43	775	1168	0.657	2.5623	48	1862	2798.5	0.6825	3.48075
rpl33	124	66994	67470	45	758	660	1.014	20	4168.5	3840.5			27	623	2584	0.538	2.7438
rps18	125	67281	68186	48	2563	804.5	3.966	38	586.5	2050	0.8535	3.32865	38	2346.5	4177.5	0.4215	2.14965
rpl20	126	68050	68630	48	1086.5	887	3.4445	34	1431.5	990.5	0.8905	3.47295	40	866.5	3221.5	0.6675	3.40425
rpl20-IG	127	68261	68930	48	628	146.5	2.5685	24	2809.5	3773.5	1.038	4.0482	33	589	2146	0.9	4.59
IG-rps12ex1	128	68793	69460	48	1673	1086.5	2.715	39	824	1038	0.84	3.276	46	755.5	1032.5	0.611	3.1161
rps12ex1-clpP	129	69461	70160	48	983.5	632	2.7485	26	1830.5	639	0.938	3.6582	47	778	2348	0.488	2.4888
clpP-1	130	69544	70394	42	543.5	844.5	0.7895	20	9963.5	10491.5			35	1095	6450	0.339	1.7289

				mature (long-day conditions)				greening					etiolated				
probe name	position on array	start of probe[1]	end of probe[1]	spot count[2]	Median (F635 Median - B635)[3]	Median (F532 Median - B532)[3]	Membrane enrichment value[4]	spot count[2]	Median (F635 Median - B635)[3]	Median (F532 Median - B532)[3]	Median (Median of Ratios (635/532))[4]	Membrane enrichment value[4]	spot count[2]	Median (F635 Median - B635)[3]	Median (F532 Median - B532)[3]	Median (Median of Ratios (635/532))[4]	Membrane enrichment value[4]
clpP-2	131	69755	69281	48	6077.5	1252	7.3205	42	4770.5	1686	1.235	4.8165	48	3252	1678.5	1.228	6.2628
IG-psbB	132	70265	71133	48	6555.5	2036.5	6.486	42	2827.5	1377	1.479	5.7681	48	3101.5	1446	1.5915	8.11665
psbB-1	133	70762	71780	48	14428	1892.5	7.585	42	9660.5	5297	1.381	5.3859	48	6027	8603	1.582	8.0682
psbB-2	134	71115	71608	48	3220	591.5	4.64	42	1480.5	1406.5	0.9995	3.89805	48	3409	10489.5	1.084	5.5284
psbBTN	135	71603	72640	48	7593	830.5	6.1975	42	3815	2348.5	1.2835	5.00565	48	4004.5	6081	1.049	5.3499
psbBTNH	136	71953	72823	47	4503	3159	2.91	42	2152.5	1301.5	1.352	5.2728	48	3396	2881.5	1.0295	5.25045
psbH	137	72695	73150	48	2488.5	1016	1.53	42	2598	1879.5	1.069	4.1691	48	2521	3145.5	0.78	3.978
psbH-petBex1	138	72710	73314	48	9019.5	2180.5	10.309	42	6980.5	1305.5	2.3905	9.32295	48	4844	2911.5	1.51	7.701
psbH-petBex2-in	139	72929	73848	12	2501	2803.5		12	863	600			12	952.5	478		
petBfull	140	73085	74566	48	5653.5	1835	5.7885	42	4501	2429	1.0555	4.11645	48	2780.5	3409.5	0.697	3.5547
petBex1-in	141	73061	73490	48	5374.5	2052	7.888	41	5947	1803	1.847	7.2033	48	4511	3710.5	1.459	7.4409
petBin		74800	75400	48	2972	841	5.506	42	1454	1813	0.8755	3.41445	48	2982.5	2380.5	0.659	3.3609
petBex1-in-ex2	142	73120	73990	48	5631.5	1677	6.6345	42	7211.5	3139	1.0755	4.19445	48	4932	5483.5	0.9015	4.59765
petBex2	143	73749	74607	12	2976	4948.5		12	1331	1350			12	2243.5	1257.5		
petBex2-petDex1-1	144	74382	74818	5	496	266		42	6504	2847.5	0.8815	3.43785	2	274.5	2432.5		
petBex2-petDex1-2	145	74421	74755	48	4676.5	3409.5	3.9395	42	4490.5	4610.5	0.926	3.6114	48	6387.5	8051	0.504	2.5704
petDex2-rpoA-1	146	74777	75895	48	3293	1156	4.4095	38	1780.5	1144.5	1.134	4.4226	48	2030.5	3399.5	0.6715	3.42465
petDex2-rpoA-2	147	75539	76501	48	2048	974.5	4.3645	28	2394.5	542.5	1.272	4.9608	48	1214.5	1479.5	1.023	5.2173
rpoA 3'	148	75991	76790	48	1135.5	525.5	4.681	45	1000	2160	0.999	3.8961	45	981	2056	1.065	5.4315
rpoA	149	76388	77170	48	2058	1042.5	2.366	22	5638.5	6358.5			48	1641.5	3754	0.4785	2.44035
rpoA-rps11	150	76837	77490	48	1133	371.5	3.9225	40	648.5	1663	0.807	3.1473	38	893.5	2815.5	0.869	4.4319
rps11-rpl36	151	77275	77945	48	1107	566.5	1.9685	23	5108	3689			41	1104	4532	0.47	2.397
rpl36-infA-rps8	152	77790	78898	48	1645.5	139.5	4.803	17	6562	6254			45	1226	2751	0.846	4.3146
infA	153	78001	78460	32	762.5	714.5	1.0125	19	6106	5089			29	349	1207	0.405	2.0655

				mature (long-day conditions)				greening					etiolated				
probe name	position on array	start of probe[1]	end of probe[1]	spot count[2]	Median (F635 Median - B635)[3]	Median (F532 Median - B532)[3]	Membrane enrichment value[4]	spot count[2]	Median (F635 Median - B635)[3]	Median (F532 Median - B532)[3]	Median (Median of Ratios (635/532))[4]	Membrane enrichment value[4]	spot count[2]	Median (F635 Median - B635)[3]	Median (F532 Median - B532)[3]	Median (Median of Ratios (635/532))[4]	Membrane enrichment value[4]
rps8	154	78400	79003	47	660	419	1.366	22	4551	6367			39	523	2288	0.59	3.009
rps8-rpl14-rpl16ex2	155	78797	79860	48	1879	474	1.469	20	9829	9902.5			39	1468	3264	0.518	2.6418
rpl14	156	78959	79481	46	1113	132.5	4.194	13	803	1289			34	1037	5447.5	0.4045	2.06295
rpl16in-ex1	157	79663	80168	33	660	547	0.966	28	1956.5	801	0.724	2.8236	23	124	420		
rpl16in	158	80002	81109	48	1341	524	3.161	34	1049.5	1963.5	0.6905	2.69295	45	1197	3797	0.801	4.0851
rpl16ex2-in	159	80002	80888	48	1092	331.5	3.1525	24	2412	3429	0.846	3.2994	43	1129	5263	0.594	3.0294
rps3	160	81003	81840	48	603.5	583.5	2.0275	23	5423	5519			32	638.5	2908	0.611	3.1161
rps19	161	82360	82720	48	844	124.5	2.6605	42	5669.5	16134	0.455	1.7745	47	821	2635	0.709	3.6159
rpl22	162	82459	81709	48	3425.5	8191	0.6455	42	12355	52122	0.293	1.1427	48	3666	24082.5	0.125	0.6375
trnH	163	82721	82881	48	6729.5	13550	0.6945	42	1385.5	1663	0.717	2.7963	48	3833.5	30808.5	0.156	0.7956
rpl2ex2	164	82901	83518	48	2255	1460.5	2.3405	26	1049.5	655	0.8185	3.19215	48	2167.5	2397	0.5165	2.63415
rpl2in	165	83356	84000	45	720	1605	0.698	40	865	1969	0.6885	2.68515	41	642	3984	0.373	1.9023
rpl2in-ex1	166	83410	84363	47	1176	1448	1.012	23	1168	1078			48	2064.5	4233	0.396	2.0196
rpl2ex1-rpl23	167	84263	84560	42	569	1218	0.467	26	1403	812.5	0.874	3.4086	34	506.5	2466.5	0.444	2.2644
rpl23	168	84411	84750	47	966	2158	0.7	28	954	1058.5	0.8715	3.39885	42	857.5	4108	0.4895	2.49645
IG rpl23-trnI-CAU	169	84667	84890	43	731	1424	0.384	42	445	6610	0.1045	0.40755	35	475	1786	0.371	1.8921
trnI-CAU	170	84852	84980	39	603	9902	0.383	22	2103.5	4753			48	524	4571	0.116	0.5916
ORF46-34-241	171	84955	85536	27	365	1588	0.21	21	3449	7890			28	136	1402	0.355	1.8105
ORF241	172	85408	86280	24	340.5	2296	0.203	22	1421.5	2778			29	275	2408	0.336	1.7136
ORF139	173	86161	86633	23	511	2009		20	1876.5	4730.5			30	273	1385	0.355	1.8105
ORF38-99	174	86534	87698	29	280	1298	0.277	23	1302	4066			29	319	1194	0.443	2.2593
ORF99	175	86911	87870	25	304	1418	0.256	25	851	898	0.616	2.4024	30	453	1081.5	0.426	2.1726
ORF99-137	176	87599	88430	30	405.5	2107.5	0.2025	12	1240.5	2040			39	357	1485	0.393	2.0043
IG ORF137-trnL-GAC	177	88381	88570	21	114	1440		41	528	9336	0.095	0.3705	15	46	509		
trnL-GAC	178	88568	88674	48	287	3674.5	0.169	36	2094.5	1027.5	0.829	3.2331	48	877.5	7987.5	0.0845	0.43095
IG-ndhBex2	179	88661	89658	48	2263	1284.5	4.4065	39	1387	1260	0.841	3.2799	48	1174	3093	0.679	3.4629

				mature (long-day conditions)				greening					etiolated				
probe name	position on array	start of probe[1]	end of probe[1]	spot count[2]	Median (F635 Median - B635)[3]	Median (F532 Median - B532)[3]	Membrane enrichment value[4]	spot count[2]	Median (F635 Median - B635)[3]	Median (F532 Median - B532)[3]	Median (Median of Ratios (635/532))[4]	Membrane enrichment value[4]	spot count[2]	Median (F635 Median - B635)[3]	Median (F532 Median - B532)[3]	Median (Median of Ratios (635/532))[4]	Membrane enrichment value[4]
ndhBex2	180	89201	90020	48	2377.5	1191	5.335	21	2907	4779			48	2312.5	4752	0.5675	2.89425
ndhBin-2	181	89552	90145	47	577	828	0.756	33	1841	3040	0.754	2.9406	40	419.5	1808	0.5525	2.81775
ndhBex2-in	182	89930	90720	37	2753	395	7.162	40	836.5	2016.5	0.6235	2.43165	37	2335	10966	0.342	1.7442
ndhBin-1	183	90044	90718	48	915.5	851	2.3795	42	3687	1435.5	1.0555	4.11645	45	938	3470	0.594	3.0294
ndhBex1	184	90618	91590	48	2641.5	1710	7.4135	41	540	1705	0.713	2.7807	48	2409	4795.5	0.633	3.2283
ndhB-rps7	185	91441	91820	48	1441	1038.5	4.3945	38	657	1303.5	0.6815	2.65785	48	1514.5	2816	0.5595	2.85345
rps7	186	91735	92225	48	921.5	677.5	3.9465	44	917	2038.5	0.8145	3.17655	45	1532	8354	0.436	2.2236
rps7-rps12ex3-in2	187	92164	92690	48	2688	1686	6.587	42	1066	2276.5	0.7475	2.91525	48	2904.5	6125	0.661	3.3711
rps12ex3-in-ex2	188	92226	93201	48	2287	1379.5	2.3955	37	969	2032	0.698	2.7222	48	2001.5	4241	0.581	2.9631
rps12ex3-in	189	92281	92860	48	2092	1217.5	3.772	24	4806	4735	0.9635	3.75765	44	1477	4852	0.5055	2.57805
rps12ex2-in1	190	92876	93570	48	1477	648	3.214	42	739.5	1491	0.7385	2.88015	42	956	5454.5	0.388	1.9788
IGrps12-ORF58-1	191	93161	94190	48	2540.5	1378	2.628	19	1916	3227			48	2251.5	3037.5	0.5735	2.92485
IGrps12-ORF58-2	192	94041	94285	24	280.5	978	0.3335	22	6515.5	11575			26	189	1305	0.396	2.0196
ORF58	193	94236	94790	27	838	1968	0.288	41	626	8842	0.195	0.7605	28	586.5	1256	0.1755	0.89505
trnV-GAC-2	194	94751	94946	40	741.5	18436.5	0.287	41	41993	55410	0.948	3.6972	48	1122	8133	0.088	0.4488
trnV-GAC-16S	195	94931	95656	48	44260	24047	1.0065	45	7880	17905	0.597	2.3283	48	24691.5	57426.5	0.6195	3.15945
16S-1	196	95560	96780	48	11150.5	6593	2.4445	42	7429	12939.5	0.5935	2.31465	48	8899	13625.5	0.447	2.2797
16S-2	197	96441	96950	48	11109.5	4993	2.2355	42	7176	7914.5	0.523	2.0397	48	6697	9355	0.519	2.6469
16S-3	198	96683	97930	48	10543.5	9257	3.187	42	1183	2525	0.419	1.6341	48	5341.5	9632	0.342	1.7442
16S-trnI-GAUex1-in	199	96953	97540	48	3168	1298.5	3.0815	42	3247.5	6903.5	0.4675	1.82325	48	1769	5849	0.3165	1.61415
trnI-GAUex1-in	200	96956	97973	48	3650.5	6656.5	2.4645	42	6282.5	6035.5	0.531	2.0709	48	3768.5	11238	0.21	1.071
trnI-GAU full	201	96991	97930	48	10573	10852	3.7875	42	4716	6790	0.454	1.7706	48	5710.5	12558.5	0.321	1.6371
trnI-GAUin-ex2-trnA-UGCex1-in	202	97814	98644	48	6296	8801	2.7965	45	2164	5459	0.407	1.5873	48	5201.5	11396	0.251	1.2801

				mature (long-day conditions)				greening					etiolated				
probe name	position on array	start of probe[1]	end of probe[1]	spot count[2]	Median (F635 Median - B635)[3]	Median (F532 Median - B532)[3]	Membrane enrichment value[4]	spot count[2]	Median (F635 Median - B635)[3]	Median (F532 Median - B532)[3]	Median (Median of Ratios (635/532))[4]	Membrane enrichment value[4]	spot count[2]	Median (F635 Median - B635)[3]	Median (F532 Median - B532)[3]	Median (Median of Ratios (635/532))[4]	Membrane enrichment value[4]
trnA-UGC full	203	98038	98915	48	3994	5179.5	2.4705	42	1543.5	2613.5	0.5345	2.08455	48	3391.5	11603	0.2155	1.09905
trnA-UGC in	204	98079	98644	48	3120.5	1524	3.1025	42	7673	12924.5	0.5045	1.96755	48	2617.5	4746	0.4495	2.29245
trnA-UGCin-ex2	205	98333	99792	48	14261.5	4680	3.072	42	26366.5	57281	1.0585	4.12815	48	4384.5	6928	0.431	2.1981
trnA-UGCin-ex2-23S	206	98711	99060	48	25277	19058.5	3.26	42	43651	57845	1.06	4.134	48	22098.5	21428.5	1.088	5.5488
23S-1	207	99067	100618	48	61880	11452.5	2.377	42	37098	28337	1.059	4.1301	48	45391	38673.5	0.9895	5.04645
23S-2	208	100098	101639	48	48085	16484	2.3545	42	31345	21195	1.061	4.1379	48	32412.5	40812	1.066	5.4366
23S-3	209	101126	102060	48	47356	16972.5	0.987	42	3547.5	1933.5	1.626	6.3414	48	28594	40078.5	1.221	6.2271
4.5S	210	101951	102270	48	5357	2065.5	2.8415	42	25701	9125	1.865	7.2735	48	4177	5464	1.2215	6.22965
5S	211	102161	102619	48	18351.5	6669	2.696	42	18281.5	8649.5	2.3375	9.11625	48	10602.5	23824	0.8525	4.34775
5S-IG	212	102363	102740	48	15934.5	9114.5	2.644	38	681.5	1212	0.481	1.8759	48	10034.5	17763	0.883	4.5033
trnR-ACG	214	102631	103061	48	487.5	834.5	0.664	42	10625.5	38955	0.338	1.3182	48	740	4297	0.1755	0.89505
trnN-GUU	215	102941	103450	48	5949	12893	0.77	35	1105	2003	0.589	2.2971	48	10807.5	19420.5	0.1925	0.98175
IG-ORF63	216	103203	104382	37	2173	559	5.101	33	4529	1003	1.077	4.2003	36	1232.5	4198.5	0.4805	2.45055
ORF63-IG	217	104004	104750	48	1158	972.5	4.8195	42	932	1494	0.921	3.5919	44	1477	3117.5	0.6235	3.17985
rps15	218	104382	105057	48	1486.5	1095	3.059	30	2419.5	765	1.2435	4.84965	47	1342	3405	0.586	2.9886
rps15-ndhF	219	104954	105217	48	1604	474.5	2.2	29	3898	1532	1.283	5.0037	48	1034.5	909	0.763	3.8913
ndhF 3'	220	105071	106310	48	917.5	240.5	5.5265	45	774	900	1.039	4.0521	42	841	1475.5	1.0135	5.16885
ndhF 5'	221	106209	107659	48	2303	1174	6.246	42	1475	3330	0.4745	1.85055	46	1496	3339.5	0.813	4.1463
ndhF-rpl32	222	107161	108424	48	2852	1554	4.4445	34	1600	837.5	1.639	6.3921	48	2388.5	2093.5	0.6135	3.12885
rpl32	223	107961	108800	48	761	560.5	2.9985	42	7478	46191.5	0.221	0.8619	38	525	1395.5	1.022	5.2122
trnL-UAG	224	108641	108930	48	2389.5	31310.5	0.284	38	1648.5	1236	1.1425	4.45575	48	5537	33536	0.113	0.5763
ccsA	225	108881	110021	48	1254	1247	3.23	42	4850.5	1984	1.694	6.6066	48	925.5	1444	0.5415	2.76165
ccsA 3'	226	109421	110130	48	2357.5	1088	3.1725	28	3646.5	1004	1.4275	5.56725	47	1002	1214	0.782	3.9882
ndhD 3'	227	110021	110615	48	3242	1030.5	4.9255	21	4169	4385			45	1881	2893	0.828	4.2228
ndhD	228	110495	111610	48	1236.5	622	3.3	44	3053	1260	1.162	4.5318	35	685	1181	0.809	4.1259
ndhD 5'	229	110661	111740	48	3294.5	1580.5	5.661	42	1634	3159	0.7545	2.94255	48	1580.5	2268	1.0035	5.11785
ndhD-psaC	230	111509	112226	48	3880.5	2946.5	2.5035	42	8503.5	10381.5	0.503	1.9617	48	3412	7589.5	0.5065	2.58315

				mature (long-day conditions)				greening					etiolated				
probe name	position on array	start of probe[1]	end of probe[1]	spot count[2]	Median (F635 Median - B635)[3]	Median (F532 Median - B532)[3]	Membrane enrichment value[4]	spot count[2]	Median (F635 Median - B635)[3]	Median (F532 Median - B532)[3]	Median (Median of Ratios (635/532))[4]	Membrane enrichment value[4]	spot count[2]	Median (F635 Median - B635)[3]	Median (F532 Median - B532)[3]	Median (Median of Ratios (635/532))[4]	Membrane enrichment value[4]
psaC	231	111760	112020	48	4163	3017	3.5275	34	6677	7447.5	0.8755	3.41445	48	6548.5	6555.5	0.531	2.7081
IG psaC-ndhE	232	111908	112470	37	8871	1359	6.496	39	580	1797	0.656	2.5584	38	5347	15648	0.33	1.683
ndhE-ndhG	233	112660	113160	48	1751	777	4.2595	32	2617.5	1028.5	1.2425	4.84575	43	1728	6143	0.521	2.6571
ndhG 3'	234	112880	113273	48	2177.5	658.5	3.161	26	4929.5	1001	1.12	4.368	44	1944	2697	1.0455	5.33205
ndhG-IG	235	113158	113735	48	1601.5	499.5	3.052	35	3000	919	1.174	4.5786	45	1537	2744	1.059	5.4009
IG-ndhI	236	113471	114240	48	1850	738.5	5.91	42	1403.5	1361	1.111	4.3329	46	1897	2988	0.997	5.0847
ndhAex2	237	114196	114891	48	3227.5	1351	3.432	22	4237	3619			47	3151	5607	1.008	5.1408
ndhAex2-in	238	114341	115730	44	1225.5	781	1.473	38	959	2512	0.8675	3.38325	38	1009.5	1388.5	1.158	5.9058
ndhAin	239	114941	115914	48	1345	1233.5	3.0295	22	6151.5	6330			45	1965	3792	0.651	3.3201
ndhAin-ex1	240	115620	116210	48	762.5	335.5	2.6135	21	3020	3728			31	569	2062	0.95	4.845
ndhAex1-ndhH	241	115951	117067	41	652	127	3.526	39	1492	1359	1.088	4.2432	31	337	559	1.008	5.1408
ndhH	242	116363	117650	48	2038.5	513.5	3.634	32	2165.5	608.5	1.153	4.4967	45	1564	2244	0.813	4.1463
ndhH-rps15	243	116955	117800	48	1403.5	496	2.015	41	6718	9465	0.736	2.8704	44	997.5	2508	0.8345	4.25595
rps19-psbA	244	140277	336	48	6730.5	6236.5	2.1705	0					48	9249.5	17419.5	0.4125	2.10375

1 genome position on maize chloroplast chromosome (accession No. NC_001666)

2 each probe is spotted with 12 replications per chip, which leads to a maximum of 48 spots per probe across all four replicates. Probes with less than 24 detectable spots were not considered in the analysis and thus appear as gaps in the graphs in figure 2a.

3 (F635 Median - B635) = the median spot pixel intensity at wavelength 635 nm with the median background subtracted. This calculation is performed by the GenePix Pro 7.0 software (Axon Instruments) for each spot on the arrays. We then calculate the median of all the (F635 Median - B635) values, which is maximum 48 spots across all four biological replicate experiments. The same applies to the signal in the other wavelength analyzed (Median (F532 Median - B532).

4 in case of data from mature chloroplasts, the MEV and the Median (Median of Ratios (635/532)) are identical. In case of greening and etiolated plastids, the MEV is derived from the Median (Median of Ratios (635/532)) by normalization against the Median (Median of Ratios (635/532)) from experiments with mature chloroplasts (against the sum of all Median (Median of Ratios (635/532)) values of all probes on the arrays that have a signal above background). The Median of Ratios (635/532) itself is calculated by the GenePix Pro 7.0 software (Axon Instruments) by taking the median of all the red (635 nm) over green (532 nm) ratios of each pixel within a spot area. Afterwards, the median of this Median of Ratios (635/532) of all spots across the four replicate microarray analyses is calculated. Since we have included four microarray experiments each with 12 replicate spots for each PCR product, this amounts maximum to 48 spots per PCR product.
