# Supplementary material for: Stable Membrane-Association of mRNAs in Etiolated, Greening and Mature Plastids
Source: Int J Mol Sci. 2017 Aug 31;18(9):1881. doi: 10.3390/ijms18091881 (PMC5618530; doi:10.3390/ijms18091881)
Supplement: Supplementary file 1 [file ijms-18-01881-s001.zip › Table S2.pdf]

Supplemental Table 2: Top-most and least enriched RNAs in chloroplast membrane preparations

long-day conditions

TOP 10 % probes			Bottom 10 % probes		
Probe name	spot count[1]	Membrane enrichment value[2]	Probe name	spot count[1]	Membrane enrichment value[2]
psaA	48	12.74	trnVin-ex1-IG	47	0.469
rps14	48	12.4585	rpl2ex1-rpl23	42	0.467
psaB-psaA	48	12.3	trnC IG	48	0.4545
psaB-1	48	12.043	trnF	48	0.439
psbD 5'	47	11.89	IG-trnC-IG	29	0.435
cemA-petA	48	11.801	IG-trnT-GGU	39	0.394
psbZ	48	11.53	trnE-UCC	48	0.3885
rps14-psaB	48	11.4355	IG rpl23-trnI-CAU	43	0.384
ycf4-cemA	48	11.2605	trnI-CAU	39	0.383
psbH-petBex1	48	10.309	IG-trnT-GGU-IG	39	0.379
atpB 5'	36	9.0125	IGrps12-ORF58-2	24	0.3335
psaB-2	48	8.0185	rpoC2-1	24	0.3325
ycf4	48	7.985	trnE-UCC-trnY-GUA	48	0.3155
petBex1-in	48	7.888	ORF58	27	0.288
psbB-1	48	7.585	trnV-GAC-2	40	0.287
ndhBex1	48	7.4135	trnL-UAG	48	0.284
clpP-2	48	7.3205	ORF38-99	29	0.277
ndhBex2-in	37	7.162	ORF99	25	0.256
psbD-psbC	48	7.0435	trnQ	48	0.252
petA	48	6.913	ORF46-34-241	27	0.21
petBex1-in-ex2	48	6.6345	ORF241	24	0.203
rps7-rps12ex3-in2	48	6.587	ORF99-137	30	0.2025
IG psaC-ndhE	37	6.496	trnL-GAC	48	0.169

Colour code
- Photosystem I
- Photosystem II
- Cytochrome b_6f complex
- NADH dehydrogenase
- ATP Synthase
- mixed probes
- ribosomal proteins
- tRNAs
- rRNAs
- Open reading frames with unknown functions
- miscellaneous genes

de-etiolated

TOP 10 % probes			Bottom 10 % probes		
Probe name	spot count[1]	Membrane enrichment value[2]	Probe name	spot count[1]	Membrane enrichment value[2]
psbH-petBex1	42	9.32295	petN	42	1.2948
5S-IG	42	9.11625	trnG-GCC	40	1.26945
psaB-psaA	42	7.72785	trnW-trnP	42	1.2597
rps14	41	7.5036	trnR-ACG-2	42	1.24605
5S	42	7.2735	trnG-UCC full	42	1.2168
psaB-1	42	7.2072	trnfM	42	1.14465
petBex1-in	41	7.2033	trnH	42	1.1427
cemA-petA	42	6.8913	trnM	41	1.1232
ccsA 3'	42	6.6066	trnT-UGU	42	1.12125
rpl32	34	6.3921	trnS-UGA	42	1.09395
4.5S	42	6.3414	IG-trnT-GGU	41	1.0452
psaA	42	6.3258	IG-trnT-GGU-IG	26	0.9789
cemA	42	5.95725	trnL-UAG	42	0.8619
psaA-3'	42	5.9553	trnQ	39	0.8541
psbD-psbC	42	5.8578	trnE-UCC-trnY-GUA	36	0.7644
IG-psbB	42	5.7681	trnV-GAC-2	41	0.7605
ndhD 3'	28	5.56725	trnE-UCC	42	0.6825
rps14-psaB	42	5.5653	trnF	40	0.4485
psbB-1	42	5.3859	trnI-CAU	42	0.40755
rps2	30	5.34495	trnL-GAC	41	0.3705

Colour code

- Photosystem I
- Photosystem II
- Cytochrome b_6f complex
- NADH dehydrogenase
- ATP Synthase
- mixed probes
- ribosomal proteins
- tRNAs
- rRNAs
- Open reading frames with unknown functions
- miscellaneous genes

etiolated

TOP 10 % probes			Bottom 10 % probes		
Probe name	spot count[1]	Membrane enrichment value[2]	Probe name	spot count[1]	Membrane enrichment value[2]
psaB-psaA	48	10.84005	ORF69-IG	24	0.93075
psaB-1	48	8.9199	trnF	48	0.918
cemA-petA	48	8.7516	ORF58	28	0.89505
psaA	48	8.619	trnR-ACG-2	48	0.89505
IG-psbB	48	8.11665	trnL-UAAin-ex2-IG	48	0.82875
psaA-3'	48	8.0937	trnT-UGU	47	0.8007
psbD-psbC	48	8.0784	trnH	48	0.7956
psbB-1	48	8.0682	trnS-UCC-rps4-2	47	0.7752
ycf4-cemA	48	7.72395	trnT-UGU-IG	48	0.7242
psbH-petBex1	48	7.701	IG-trnL-UAAex1	48	0.714
psaB-2	48	7.6041	trnM	48	0.69615
petBex1-in	48	7.4409	trnS-UGA	48	0.6834
rps14	48	7.40265	trnT-GGU	24	0.64515
cemA	48	7.3389	trnG-UCC full	48	0.6426
psbD 5'	48	6.93345	rpl22	48	0.6375
IG-atpH	48	6.6249	trnI-CAU	48	0.5916
rps14-psaB	48	6.31635	trnL-UAG	48	0.5763
clpP-2	48	6.2628	trnQ	48	0.5304
4.5S	48	6.22965	trnW-trnP	48	0.5253
23S-3	48	6.2271	trnE-UCC-trnY-GUA	48	0.5202
rps2	44	5.9874	trnC IG	48	0.51765
ndhAex2-in	38	5.9058	trnE-UCC	47	0.4896
atpB	47	5.8854	trnV-GAC-2	48	0.4488
psbZ	48	5.7681	trnL-GAC	48	0.43095

Colour code
- Photosystem I
- Photosystem II
- Cytochrome b_6f complex
- NADH dehydrogenase
- ATP Synthase
- mixed probes
- ribosomal proteins
- tRNAs
- rRNAs
- Open reading frames with unknown functions
- miscellaneous genes

1 each probe is spotted with 12 replications per chip, which leads to a maximum of 48 spots per probe across all four replicates. Probes with less than 24 detectable spots were not considered int he analysis and thus appear as gaps in the graphs in figure 3.

2 in case of data from mature chloroplasts, the MEV and the Median (Median of Ratios (635/532)) are identical. In case of greening and etiolated plastids, the MEV is derived from the Median (Median of Ratios (635/532)) by normalization against the Median (Median of Ratios (635/532)) from experiments with mature chloroplasts (against the sum of all Median (Median of Ratios (635/532)) values of all probes on the arrays that have a signal above background). The Median of Ratios (635/532) itself is calculated by the GenePix Pro 7.0 software (Axon Instruments) by taking the median of all the red (635 nm) over green (532 nm) ratios of each pixel within a spot area. Afterwards, the median of this Median of Ratios (635/532) of all spots across the four replicate microarray analyses is calculated. Since we have included four microarray experiments each with 12 replicate spots for each PCR product, this amounts maximum to 48 spots per PCR product.
