# Supplementary material for: Stable Membrane-Association of mRNAs in Etiolated, Greening and Mature Plastids
Source: Int J Mol Sci. 2017 Aug 31;18(9):1881. doi: 10.3390/ijms18091881 (PMC5618530; doi:10.3390/ijms18091881)
Supplement: Supplementary file 1 [file ijms-18-01881-s001.zip › Table S3.pdf]

Table S3: 20% least membrane-enriched mRNAs in chloroplast membrane preparations

mature			greening			etiolated		
Probe name	spot count[1]	Membrane enrichment value[2]	Probe name	spot count[1]	Membrane enrichment value[2]	Probe name	spot count[1]	Membrane enrichment value[2]
orf99-137	30	0.2025	petN	42	1.2948	rpl22	48	0.6375
orf241	24	0.203	atpA-3'	42	1.40205	orf58	28	0.89505
orf46-34-241	27	0.21	petN-IG	37	1.404	orf69-IG	24	0.93075
orf99	25	0.256	petG	42	1.69065	petG	48	1.00725
orf38-99	29	0.277	IG psbM-petN	36	1.76085	matK	26	1.08885
orf58	27	0.288	rpl22	42	1.7745	petL	37	1.1934
rpoC2-1	24	0.3325	IG-rps16 ex2	42	1.79985	rpoC2-1	26	1.2189
IGrps12-orf58-2	24	0.3335	rbcL 5'	42	1.8174	psaJ	37	1.2648
rpl2ex1-rpl23	42	0.467	ndhF-rpl32	42	1.85055	psbA 3'	48	1.27245
rpl22	48	0.6455	rps16 full	42	1.9305	IG-rps16 ex2	48	1.37445
rpl2in	45	0.698	psbM-IG	30	1.94805	psbA 5'	48	1.54275
rpl23	47	0.7	IG-psaJ-IG	42	1.95975	trnM-atpE-atpB	26	1.58865
matK	24	0.756	psaC	42	1.9617	IG psaC-ndhE	38	1.683
ndhBin-2	47	0.756	rbcL	40	1.98315	orf241	29	1.7136
clpP-1	42	0.7895	atpB	39	2.2035	psbM-IG	38	1.72125
rbcL	48	0.7995	psbJLFE	42	2.27955	clpP-1	35	1.7289
rps16 in	45	0.87	IG-orf63	35	2.2971	ndhBex2-in	37	1.7442
rpl16in-ex1	33	0.966	psbA	36	2.38875	atpA-3'	48	1.75695
psbA 5'	47	0.987	IG-psbK	42	2.39265	IG-psaJ-IG	48	1.79775
psbA	48	0.988	orf99-137	25	2.4024	orf139	30	1.8105
rpl2in-ex1	47	1.012	rbcL 3'	37	2.4063	orf46-34-241	28	1.8105
infA	32	1.0125	ndhBin-1	40	2.43165	petN-IG	47	1.887
rpl33	45	1.014	psbA 3'	42	2.43165	rpl2in	41	1.9023
IG psbM-petN	48	1.016	psaJ	28	2.52135	petN	48	1.9431
IG-psaI-1	24	1.0275	IG atpB-rbcL	42	2.54475	rps12ex2-in1	42	1.9788
petN-IG	48	1.133	ndhE-ndhG	39	2.5584	orf99-137	39	2.0043
atpA-5'	48	1.1445	psaJ-rpl33	43	2.5623	IGrps12-orf58-2	26	2.0196
petN	48	1.2615	rps16 in	27	2.574	rpl2in-ex1	48	2.0196
petG	48	1.3605				psbA	48	2.04
rps8	47	1.366				rpl14	34	2.06295
atpFex1-2	48	1.4465				infA	29	2.0655
rps8-rpl14-rpl16ex2	48	1.469				rps19-psbA	48	2.10375
ndhAex2-in	44	1.473				atpB 5'	36	2.13945
psbJ	48	1.482				rps18	38	2.14965

Colour code

- Photosystem I
- Photosystem II
- Cytochrome b_6f complex
- NADH dehydrogenase
- ATP Synthase
- mixed probes
- ribosomal proteins
- tRNAs
- rRNAs
- Open reading frames with unknown functions
- miscellaneous genes

1 each probe is spotted with 12 replications per chip, which leads to a maximum of 48 spots per probe across all four replicates. Probes with less than 24 detectable spots were not considered int he analysis and thus appear as gaps in the graphs in figure 2A.

2 in case of data from mature chloroplasts, the MEV and the Median (Median of Ratios (635/532)) are identical. In case of greening and etiolated plastids, the MEV is derived from the Median (Median of Ratios (635/532)) by normalization against the Median (Median of Ratios (635/532)) from experiments with mature chloroplasts (against the sum of all Median (Median of Ratios (635/532)) values of all probes on the arrays that have a signal above background). The Median of Ratios (635/532) itself is calculated by the GenePix Pro 7.0 software (Axon Instruments) by taking the median of all the red (635 nm) over green (532 nm) ratios of each pixel within a spot area. Afterwards, the median of this Median of Ratios (635/532) of all spots across the four replicate microarray analyses is calculated. Since we have included four microarray experiments with each carrying 12 replicate spots of each PCR product, this amounts maximum to 48 spots.
