# Supplementary material for: Stable Membrane-Association of mRNAs in Etiolated, Greening and Mature Plastids
Source: Int J Mol Sci. 2017 Aug 31;18(9):1881. doi: 10.3390/ijms18091881 (PMC5618530; doi:10.3390/ijms18091881)
Supplement: Supplementary file 1 [file ijms-18-01881-s001.zip › Table S4.pdf]

Table S4: List of oligonucleotides

Name	Sequence (5' to 3')	Purpose
Ck_psbD_for	CTATTTCGCTTTAGGGGGTTGG	riboprobe for *psbD*
Ck_psbD_T7_rev	TAATACGACTCACTATAGGGCAGCAATGGGACCAGAGAATGC	
SK psbAex1forw.	ACCGTCTTTACATTGGATGG	riboprobe for *psbA*
psbA_zm.T7	GTAATCGACTCACTATAGGGTACCAGATATTCCTAAAGGC	
psbB.rp	TTTGCAGCTTTTGTTGTTGC	riboprobe for *psbB*
psbB.T7	GTAATACGACTCACTATAGGGCTTCTAAACGGGACGTCAGC	
petA.43.for	CTCGCTCCATATCTGTCTCACTC	riboprobe for *petA*
petA.381.T7.rev	TAATACGACTCACTATAGGGCTCGGGTGAAATACGATCG	
CK_rps16_for	CGATGTGGTAGAAAGCAACG	riboprobe for *rps16*
CK_rps16_T7	GTAATACGACTCACTATAGGGCATTCCGTAAAAATCCCAGC	
Zmrrn16fw	GCTCAGGATGAACGCTGGCG	double-stranded DNA probe for *rrn16*
Zmrrn16rev	CATCCTCTGTCTTACCGCGGC	
psaC.rp	CTTGTATAGGATGTACTCAATGTGTC	riboprobe for *psaC*
psaC.T7	GTAATCGACTCACTATAGGGCCATACTTCGAGTTGTTTCATGC	
Zmrrn5fw	TATTCTGGTGTCCTAGGCGTAG	double-stranded DNA probe for *rrn5*
Zmrrn5rev	ATTCTGGCATCGAGCTATTTTGC	
CK_rbcL_for	GCAGCATTCCGAGTAACTCC	riboprobe for *rbcL*
CK_rbcL_T7	GTAATACGACTCACTATAGGGCCACGTAGACATTCATAAACTGC	
Zm_atpHfor	TTTGACACGAGGAACTCATC	riboprobe for *atpH*
Zm_atpHrev_T7	GTAATACGACTCACTATAGGGCAAATAAAAGCGCTAGTGC	
